# Li_1.5_La_1.5_*M*O_6_ (*M* = W^6+^, Te^6+^) as a new series of lithium-rich double perovskites for all-solid-state lithium-ion batteries

**DOI:** 10.1038/s41467-020-19815-5

**Published:** 2020-12-15

**Authors:** Marco Amores, Hany El-Shinawi, Innes McClelland, Stephen R. Yeandel, Peter J. Baker, Ronald I. Smith, Helen Y. Playford, Pooja Goddard, Serena A. Corr, Edmund J. Cussen

**Affiliations:** 1 Department of Chemical and Biological Engineering, University of Sheffield, Sheffield, S1 3JD UK; 2 The Faraday Institution, Harwell Campus, Didcot, OX1 0RA UK; 3 Department of Chemistry, Loughborough University, Epinal Way, Loughborough, LE11 3TU UK; 4 ISIS Pulsed Neutron and Muon Source, STFC Rutherford Appleton Laboratory, Harwell Science and Innovation Campus, Didcot, Oxfordshire OX11 0QX UK; 5 Department of Materials Science and Engineering, University of Sheffield, Sheffield, S1 3JD UK

**Keywords:** Energy, Solid-state chemistry, Structure of solids and liquids, Batteries

## Abstract

Solid-state batteries are a proposed route to safely achieving high energy densities, yet this architecture faces challenges arising from interfacial issues between the electrode and solid electrolyte. Here we develop a novel family of double perovskites, Li_1.5_La_1.5_*M*O_6_ (*M* = W^6+^, Te^6+^), where an uncommon lithium-ion distribution enables macroscopic ion diffusion and tailored design of the composition allows us to switch functionality to either a negative electrode or a solid electrolyte. Introduction of tungsten allows reversible lithium-ion intercalation below 1 V, enabling application as an anode (initial specific capacity >200 mAh g^-1^ with remarkably low volume change of ∼0.2%). By contrast, substitution of tungsten with tellurium induces redox stability, directing the functionality of the perovskite towards a solid-state electrolyte with electrochemical stability up to 5 V and a low activation energy barrier (<0.2 eV) for microscopic lithium-ion diffusion. Characterisation across multiple length- and time-scales allows interrogation of the structure-property relationships in these materials and preliminary examination of a solid-state cell employing both compositions suggests lattice-matching avenues show promise for all-solid-state batteries.

## Abstract (web summary)

The development of the all solid-state battery requires the formation of stable solid/solid interfaces between different battery components. Here the authors tailor the composition to form both electrolyte and anode from the same novel family of perovskites with shared crystal chemistry.

## Introduction

Accessing high performance all-solid-state Li-ion batteries remains an outstanding grand challenge in the battery research community. The desire to move towards an all solid battery configuration is driven by safety concerns, enabling the use of metallic lithium anodes and the replacement of flammable liquid electrolytes used ubiquitously in existing Li-ion batteries^1,2^. These liquid electrolytes are readily flammable and chemically unstable at high voltages and temperatures, which compromises the safety of the battery^3–5^. All-solid-state Li batteries are a promising alternative, which could overcome not only these pressing safety concerns but would also see an increase in the achievable energy density in these Li batteries by extending the potential window to permit the safe use of high-voltage cathodes and metallic lithium anodes^6^. However, a lack of reliable solid-state electrolytes is currently hampering the development of all-solid-state batteries and complexities of delivering high Li-ion diffusion at the electrode–electrolyte interface mean that there is a pressing need for new families of functional solid-state materials to fulfil this role^7–9^.

To address this challenge, we have turned to the perovskite family of compounds, where the prototype cubic structure possesses the formula unit *AB*O_3_, with *A*-site metal cations 12-coordinated to oxygen and *B*-site cations octahedrally coordinated by 6 oxygen atoms. Oxide perovskite materials are versatile materials with an impressive range of applications due to their exotic physical properties, including ferroelectric, dielectric, pyroelectric, and piezoelectric behaviours^10^. This versatility owes much to the robust framework, which permits multiple combinations of different cations and anions to be present in the structure, making this an ideal candidate for battery applications as it can accommodate a wide range of cation sizes and oxidation states. Lower-symmetry structures can also be obtained by variations in the relative sizes of the *A* and *B* cations^10,11^. Our intention is to derive a family of compounds where judicious choice of the *B*-site cation can lead to changes in conductivity, allowing for the design of active electrode and solid-electrolyte materials of the same structure type. Our ultimate ambition is to realise a family of lithium-containing materials where chemical compatibility and ion mobility at the electrode–electrolyte boundary are maximised by shared crystal structure and minimal changes in compositional variation across this interface.

Lithium is most commonly accommodated in the perovskite structure in the oxide octahedra. However, there is an extensively studied class of compounds where lithium is introduced into the larger site to give stoichiometries such as (La,Li)TiO_3_. These materials continue to attract great interest owing to reports of fast ion conduction, e.g., in Li_0.34_La_0.5_TiO_2.94_^12,13^. This arises from displacement of the lithium from the centre of the perovskite cube to occupy a four-coordinated, distorted square-planar site. An extensive compositional search reveals that both the structural arrangement and the ionic conductivity are highly anomalous and the fast conduction of Li_0.34_La_0.5_TiO_2.94_ has not been reproduced in analogues based on replacement of La^3+^ with other rare earth cations or alkaline earth elements or by substitution of Ti^4+^ with Zr^4+^, Nb^5+^, Ta^5+^ or other appropriate cations^14–17^.

**Fig. 1:**
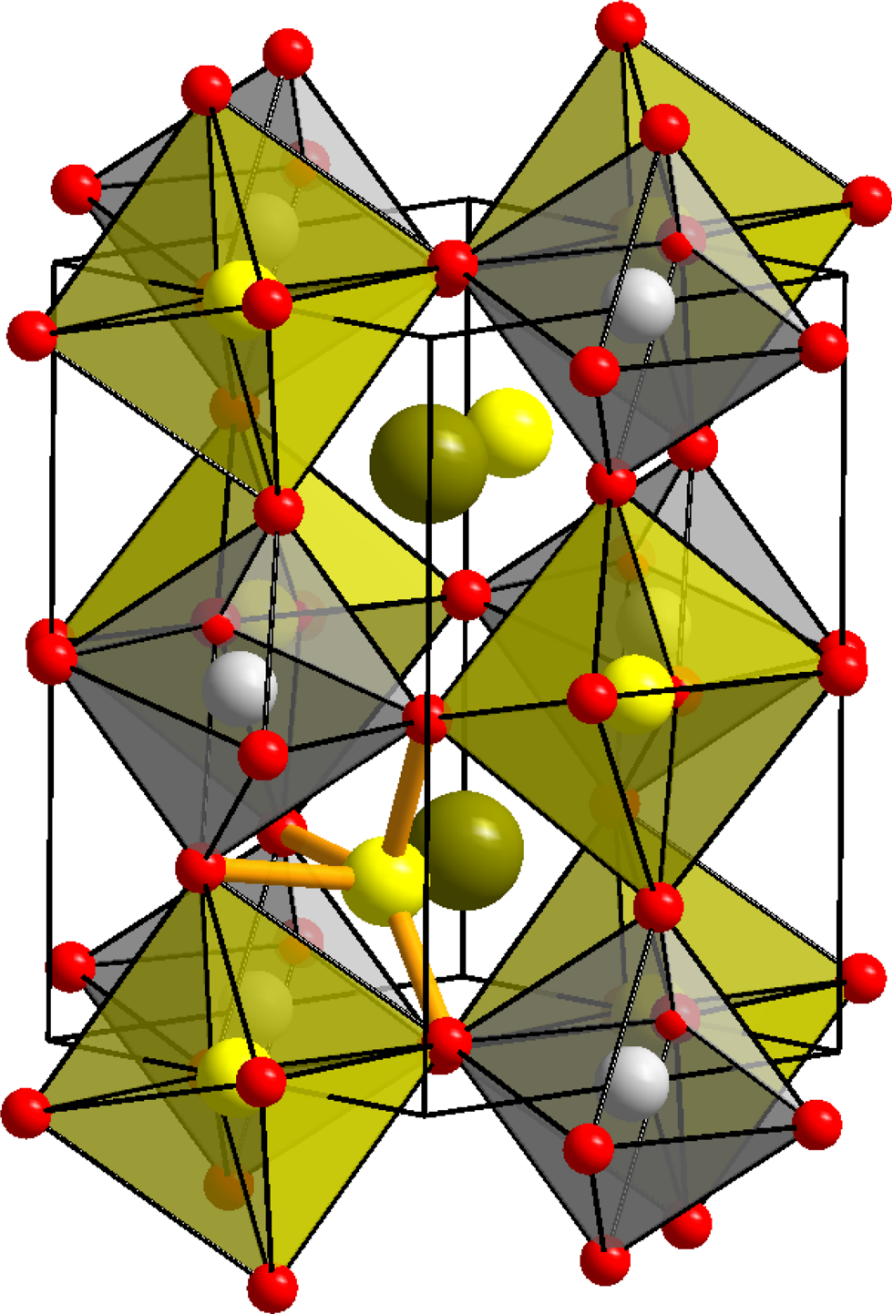
Crystal structure of Li_1.5_La_1.5_*M*O_6_ double perovskites. Cation ordering across octahedral sites occurs as shown here in the monoclinically distorted double perovskite structure of Li_1.5_La_1.5_WO_6_. Grey spheres in the octahedra represent W^6+^ ions; dark green spheres represent La^3+^ ions at the centre of the large *A*-site interstice. Lithium cations are shown as yellow spheres occupying half of the oxide octahedra in a rock salt ordering pattern. The remaining lithium cations occupy four-coordinated sites that are displaced from the centre of the large *A*-site interstice. The coordination environment for one LiO_4_ unit is indicated by orange bonds.

Despite the multiple applications of perovskite materials, their use in Li-ion batteries is limited to only a few reports, namely, lithium lanthanum titanate as a fast lithium conductor and lithium lanthanum niobate as an insertion electrode^13,18^. Introduction of a second cation on the *B* site can introduce additional complexity from chemical ordering of these cations over different crystallographic sites in so-called double perovskite structures as shown in Fig. 1^17^. We have serendipitously discovered a lithium tungstate double perovskite, which has also been independently identified by another group^19^.

Here we demonstrate for the first time that the combination of lithium mobility along with a redox active metal in a high oxidation state allows for the electrochemical (de)intercalation of lithium to provide a new class of electrode materials for Li-ion batteries. Since the perovskite structure is famously amenable to chemical and structural adjustment, we propose that this is the first in a new class of perovskite lithium electrode materials. To further demonstrate this chemical adjustment and how it pertains to property tuning, we also report a tellurium analogue, which displays promising solid-electrolyte capabilities. As well as their comprehensive physical and chemical characterisation, we also demonstrate the first proof-of-concept measurements of their combined performance in a hybrid solid-state Li-ion battery.

## Results

### Synthesis, structural characterisation and chemical composition

The syntheses of novel lithium-rich double perovskites Li_1.5_La_1.5_*M*O_6_ (where *M* = W^6+^, Te^6+^) used a microwave-assisted solid-state approach. Such synthetic methods have been developed extensively in our research group in recent years and afford faster, lower temperature routes to high quality solid-state materials^20–23^. Optimisation of the reagent stoichiometries revealed that ratios of Li:La:*M* of 1.5:1.5:1 were required for phase pure compounds (*M* = W^6+^, Te^6+^). Ratios lying outside of these values consistently delivered impure products as shown by X-ray diffraction (XRD) data in Supplementary Fig. 1. Diffraction data from three target compositions Li_1.5_La_1.5_WO_6_, Li_1.5_La_1.5_TeO_6_ and Li_1.5_La_1.5_W_0.5_Te_0.5_O_6_ could readily be indexed using a monoclinically distorted variant of the perovskite structure. This distortion commonly arises from a combination of cation ordering over the octahedral sites and a tilting of the oxide octahedral to reduce the size of the large *A*-site interstice to better match the bonding requirements of *A* cations that are smaller than optimal. Li^+^ cations are well matched in size to the *B*-site octahedral sites as shown in the double perovskites La_2_Li*M*O_6_^17,24^. The stoichiometries Li_1.5_La_1.5_*M*O_6_ can be re-written as (La_1.5_Li_0.5_)(Li*M*)O_6_ to emphasise the relation to the classic *AB*O_3_ perovskite formula. This description implies that Li^+^ cations occupy both *A* and *B* sites of the structure, and this hypothesis was tested experimentally with X-ray and neutron powder diffraction (NPD). Using these two radiations in a simultaneous refinement allows the X-ray data to locate dominant X-ray scatterers, i.e. La^3+^, W^6+^ and Te^6+^, and the neutron diffraction locates the lighter Li^+^ and O^2−^. Crucially, the complementary weightings of atomic number (X-rays) and scattering length (neutrons) provides contrast that allows us to search for vacancies in these structures.

Rietveld refinement for La_1.5_Li_1.5_WO_6_ showed that the *A* site is *ca*. ^3^/_4_ occupied by La^3+^. The monoclinic distortion reduces the La coordination number to 8, from 12 in the undistorted structure. The *A* site is also partially occupied in a disordered manner by Li^+^ ions that are displaced towards the base of a trigonal pyramid (Fig. 1). This lowers the coordination number from eight for La^3+^ to four for Li^+^, and the shortening of the Li···O distances to values more typical of the smaller Li^+^ cation. Trial refinements indicated that all oxide positions were within one esd, i.e. 1%, of being fully occupied so these were subsequently fixed at this value. All other atomic fraction parameters and thermal parameters were allowed to refine freely leading to the stoichiometry La_1.43(12)_Li_1.39(4)_W_0.97(1)_O_6_ (Supplementary Table 1), within three esd of the target stoichiometry. Elemental analyses by energy-dispersive analysis of X-rays and mass spectrometry were both in agreement with this (Supplementary Tables 2 and 3). The composition of this material will be referred to as Li_1.5_La_1.5_WO_6_. The structure of Li_1.5_La_1.5_WO_6_ was also assessed by X-ray absorption spectroscopy (XAS) using the W L_III_-edge. This showed that the average W–O bond length and the W–La distance calculated from the EXAFS were in agreement with the diffraction analysis (Supplementary Fig. 2), indicating that the crystallographic structure is representative of the local configuration.

**Fig. 2:**
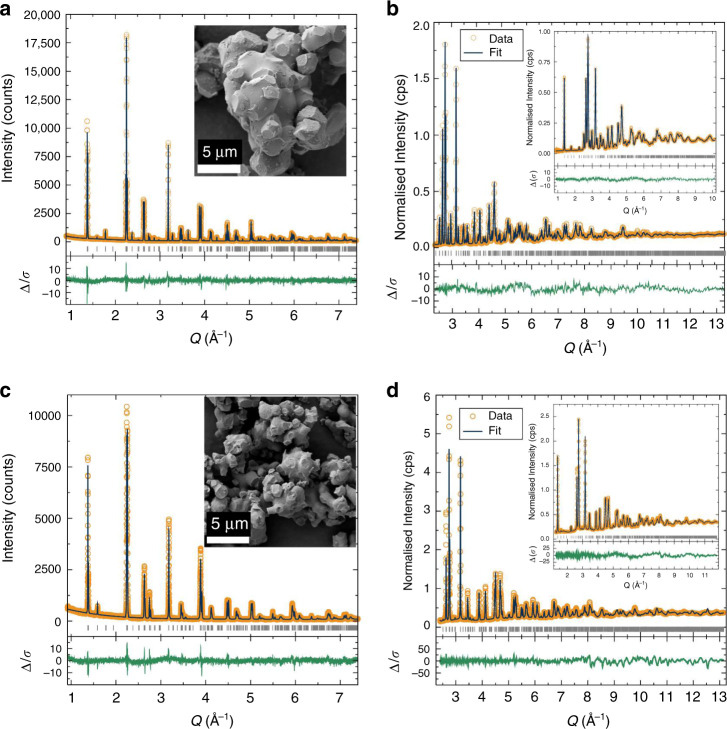
Crystal structure Rietveld refinements and particle morphology. **a** X-ray and **b** neutron powder diffraction data for the Li_1.5_La_1.5_WO_6_ material shown in the inset to (**a**). **b** Neutron diffraction data are shown over the combined Q-range 1–13 Å^−1^ using two detector banks. **c**, **d** Corresponding data from Li_1.5_La_1.5_TeO_6_. Fits were in good agreement to the monoclinic space group *P*2_1_/*n*, with a partially disordered arrangement of oxide anions in Li_1.5_La_1.5_TeO_6_. The SEM images reveal smaller particle sizes for the Te compound compared to the W analogue.

Replacement of tungsten with tellurium resulted in a material that gave X-ray and neutron diffraction profiles that could be indexed using the same *P*2_1_/*n* distortion. Simultaneous refinement of the structure against the X-ray and neutron diffraction profiles showed agreement with the structural model, but with a significant difference between the observed and calculated neutron diffraction profiles indicated by the relatively high value of *χ*^2^ = 8.4. This structural refinement was subjected to extensive testing for disorder in both Li/La and Li/Te arrangements, and insertion of Li^+^ onto additional interstitial sites; all of which indicated that the cation arrangement in Li_1.5_La_1.5_TeO_6_ was the same as the tungstate analogue, and the cation occupancies were subsequently fixed at the targeted composition. Difference Fourier searches indicated that the intensity mismatch was arising from shortcomings in the description of the oxide ions; when these displacements were modelled anisotropically, non-physical values resulted. The fit was greatly improved by allowing each of the crystallographic oxide ion positions to be split over two sites, with the fractional occupancy of each site initially refining freely. This resulted in six crystallographically independent oxide ion positions, with the occupancy of the sites showing that each position had been split in an approximate 1:2 ratio (Supplementary Fig. 3). For the final refinement, the oxide ions were paired such that a single site occupancy was used to model this disorder giving the structural parameters in Supplementary Table 4. This had negligible impact on the quality of the fit compared to the unconstrained disorder in the oxide occupancies, as shown in Fig. 2 with *χ*^2^ = 5.35. To investigate the possibility of a solid solution between both compositions, the mixed Li_1.5_La_1.5_W_0.5_Te_0.5_O_6_ double perovskite was also synthesised. XRD analysis indicates the same *P*2_1_/*n* space group, with a unit cell volume of 246.704(9) Å^3^ that is intermediate between Li_1.5_La_1.5_WO_6_ [245.016(3) Å^3^] and Li_1.5_La_1.5_TeO_6_ [247.24(1) Å^3^] (Supplementary Fig. 4). Raman spectra revealed the expected vibrational modes^25^ were present in all three compounds, with both Te–O and W–O vibrations present in the Li_1.5_La_1.5_W_0.5_Te_0.5_O_6_ material (Supplementary Fig. 5). The compositions of the three targets were further confirmed by energy-dispersive X-ray (EDX) and ICP elemental analyses (Supplementary Tables 2 and 3). We recently reported the Na-analogue Na_1.5_La_1.5_TeO_6_ indicating of the wide versatility of these novel families of alkali metal-rich double perovskites^23^. The microstructures of Li_1.5_La_1.5_WO_6_ and Li_1.5_La_1.5_TeO_6_ feature quasi-spherical particles fused together by tubular joints as shown in Fig. 2. A larger particle size was noted for the tungsten compound (5–10 μm) compared with the tellurium analogue (1–5 μm).

### Electrochemical and ion transport properties

**Fig. 3:**
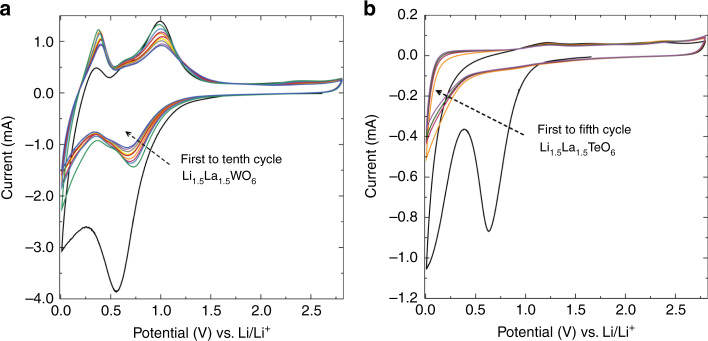
Redox response dependence of *M*^6+^ cation. **a** CV data for Li_1.5_La_1.5_WO_6_ and **b** Li_1.5_La_1.5_TeO_6_ materials mixed with 5% carbon black and 5% PTFE binder between 0.01 and 2.8 V vs Li. The scan rate was fixed at 0.1 mV s^−^^1^ for both measurements.

The presence of W^6+^ ions and the robust perovskite framework suggest this material may function as an insertion anode material, due to the accessible tungsten redox couples. To evaluate this possibility, cyclic voltammetry (CV) analyses were performed in the voltage range of 0.01–2.8 V against Li metal as the counter electrode, at a scan rate of 0.1 mV s^−^^1^. In the first cycle, a broad peak in the reduction regime is observed due to the formation of a solid-electrolyte interphase (SEI), a consequence of a partial decomposition at low voltages of the carbonate liquid electrolyte and the high surface area of the carbon black used^26,27^. Li_1.5_La_1.5_WO_6_ displays two strong redox couples (Fig. 3a). The first voltage redox couple occurs at ∼0.7 V in the reduction regime and at ∼1 V in the oxidation regime. The second redox process takes place at lower potentials; in the reduction regime the peak below 0.2 V is partially obscured by the broad peak near 0 V arising from lithium insertion into the carbon black additive^27^, and near 0.3 V for the oxidation process. Interestingly, in the case of the isostructural Li_1.5_La_1.5_TeO_6_ analogue, there are no reduction or oxidation peaks over the same potential range (Fig. 3b), apart from those arising from the carbon black additive (Supplementary Fig. 6). The intermediate phase Li_1.5_La_1.5_W_0.5_Te_0.5_O_6_ (Supplementary Fig. 7) displays similar peak positions to the W parent compound, but with reduced current presumably from lower concentration of redox active W cations.

**Fig. 4:**
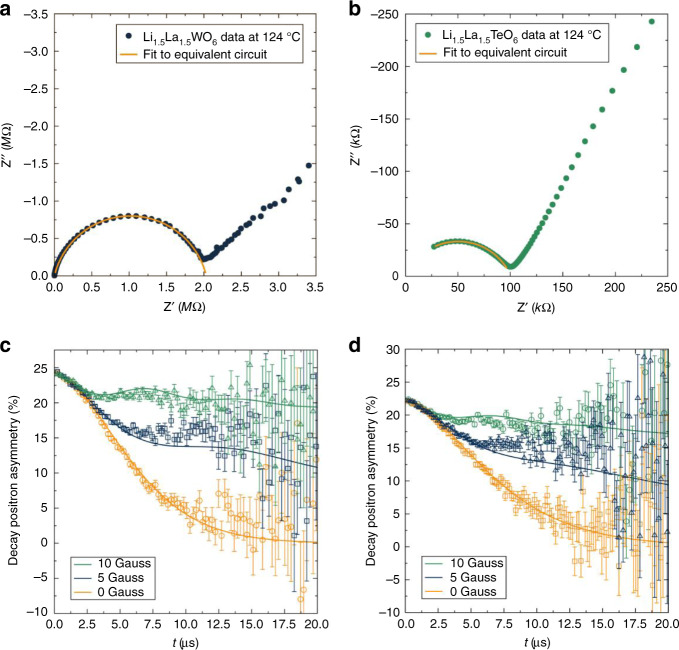
Macroscopic and local transport properties. **a** Electrical impedance for Li_1.5_La_1.5_WO_6_ and **b** Li_1.5_La_1.5_TeO_6_ at 124 °C fitted to an equivalent electrical circuit of a resistor in parallel with a constant phase element. **c** μ^+^SR experiments show the temporal evolution of the decay positron asymmetry for Li_1.5_La_1.5_WO_6_ and **d** Li_1.5_La_1.5_TeO_6_ at room temperature, with the fits to the Keren function^40^ indicated by solid lines. The error bars are indicated by raising lines on the same colour of the data set and with triangular, square or circular shapes for 10, 5 and 0 Gauss, respectively.

**Fig. 5:**
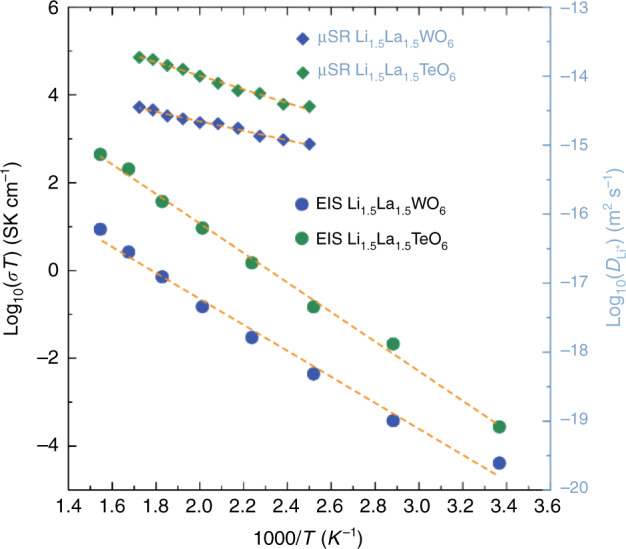
Arrhenius behaviour of conduction properties at macro and microscopic level. Arrhenius plots of the macroscopic and microscopic ionic transport properties measured by electrical impedance spectroscopy (conductivity, left axis) and μ^+^SR (diffusivity, right axis) for Li_1.5_La_1.5_WO_6_ and Li_1.5_La_1.5_TeO_6_.

The absence of redox activity in Li_1.5_La_1.5_TeO_6_ indicated that this compound could be redox stable in the potential window of a battery and suggested applications as a solid Li-ion electrolyte. Macroscopic ionic conduction was analysed for both materials by means of electrochemical impedance spectroscopy (Fig. 4), revealing activation energies for ionic conduction of 0.59(3) and 0.68(2) eV (Fig. 5) and ionic conductivities of 1.1 × 10^−5^ and 5.8 × 10^−5^ S cm^−1^ at 124 °C for the W and Te materials, respectively (Supplementary Table 5). These conductivities are intermediate between the fast conducting garnets^28–31^ based on Li_7_La_3_Zr_2_O_12_ and the lower conductivity of conventional garnets^32,33^, such as the Li_3_*Ln*_3_Te_2_O_12_, that have the same cation ratio as these perovskites. It should be noted that these conductivities derive from sintered cold pressed pellets and include contributions from both intra- and inter-grain conduction. It is widely observed across garnets and other families of ionically conducting oxides that compositional adjustments may dramatically increase intra-grain conduction, whilst inter-grain resistance may be greatly reduced by systematic adjustments to processing conditions, e.g., hot pressing or spark-plasma sintering (SPS)^34,35^. The Li-rich family of materials presented here are prime candidates for similar developments, with the added benefit that these afford both electrode and solid electrolyte with the same crystal structure and therefore present an opportunity for greater interfacial compatibility, a key consideration currently hampering the development of solid-state batteries^7,36^.

The total conductivity measured by impedance spectroscopy can be complemented by muon spin relaxation (μ^+^SR) in assessing diffusion behaviour over a local scale of a few nanometres^37^. Spin-polarised muons are implanted into the material and the spin direction is perturbed by the passage of diffusing species, such as Li^+^, that possess a nuclear moment. Analysis of the muon depolarisation as a function of temperature, combined with knowledge of the crystallographic distribution of Li^+^, allows extraction of values of room temperature Li^+^ diffusion coefficients of 6.6 × 10^−12^ cm^2^ s^−1^ and 1.8 × 10^−11^ cm^2^ s^−1^ for the W and Te materials, respectively, as shown in Figs. 4 and Supplementary Fig. 8. These values are similar to those obtained for other fast ion conductors studied using μ^+^SR, including the doped garnet materials based on Li_7_La_3_Zr_2_O_12_ (4.62 × 10^−11^ cm^2^ s^−1^), the anode material Li_4_Ti_5_O_12_ (3.2 × 10^−11^ cm^2^ s^−1^) and the high energy NMC cathode (3.5 × 10^−12^ cm^2^ s^−1^)^21,38,39^. Arrhenius analyses, shown in Fig. 5, deliver activation energies of 0.136(5) for the W compound and 0.196(8) eV for Te analogue. These values are also similar to those obtained for Na^+^ diffusion in our recently reported analogous Na-rich double perovskite, Na_1.5_La_1.5_TeO_6_, of 4.2 × 10^−12^ cm^2^ s^−1^ and 0.163(9) eV^23^, indicating the versatility of the double perovskite framework for other battery chemistries beyond lithium. Our values from μ^+^SR experiments are in agreement with DFT calculations and NMR measurements previously reported on the tungstate^19^ and are also in line with those of the related (Li,La)TiO_3_ perovskite fast ionic conductors materials (0.144 eV)^13^. These activation energies are much lower than we observe by total electrical impedance measurements for Li_1.5_La_1.5_WO_6_ and Li_1.5_La_1.5_TeO_6_. An individual muon samples a small, local region of a material and by implanting muons throughout the material samples a volume-weighted average structure. As grain boundaries make up a minority of the volume of crystalline materials, such as these perovskites, the transport properties derived from muon measurements are strongly weighted towards the intra-grain transport properties. We have seen similar effects in fast Li^+^ conducting garnet phases where the muon activation energy is much lower than that derived from impedance analysis of total conductivity^21^.

Li_1.5_La_1.5_WO_6_ shows ionic conductivity and low-voltage redox activity, suggesting possible application as an anode in Li-ion batteries. As CV measurements on Li_1.5_La_1.5_TeO_6_ showed no redox activity, galvanostatic measurements on the Te analogue were carried out in order to serve as null measurements and evaluate the capacitive contribution from the carbon black additive used to increase the electronic conductivity of the electrode.

**Fig. 6:**
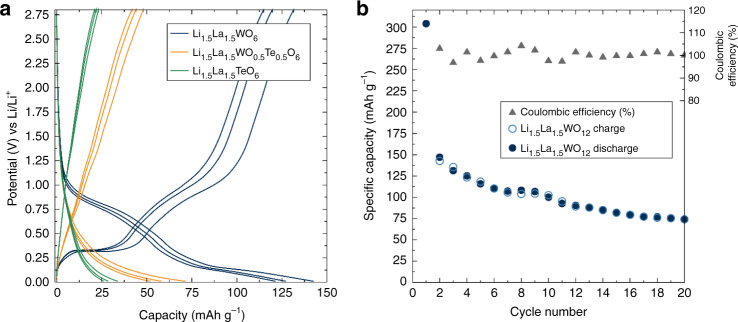
Battery cycling behaviour of Li_1.5_La_1.5_*M*O_6_ Li-rich double perovskites. **a** Galvanostatic cycling of Li_1.5_La_1.5_*M*O_6_ double perovskites mixed with 5% carbon black and 5% PTFE binder, between 0.01 and 2.8 V vs Li at a specific current of 36 mA g^−1^, where the first discharge has been omitted for clarity. **b** Charge/discharge capacity fading test for the Li_1.5_La_1.5_WO_6_ material mixed with 5% carbon black and 5% PTFE binder at a current of 36 mA g^−1^ between 0.01 and 2.8 V vs Li over 20 cycles. The initial high first discharge is due to the irreversible formation of the SEI layer caused by electrolyte decomposition.

Galvanostatic testing of Li_1.5_La_1.5_WO_6_ at a rate of 36 mA g^−1^ gave the cycling profile and discharge capacity shown in Fig. 6. The first discharge capacity is irreversibly increased to above 300 mAh g^−1^ due to electrolyte decomposition and formation of SEI. Subsequent cycles displayed reversible capacities of ∼125 mAh g^−1^ corresponding to an approximate 2e^−^ transfer per formula unit of double perovskite material after carbon capacity subtraction determined from the Li_1.5_La_1.5_TeO_6_ cycling capacity. In agreement with the CV results, a clear flat plateau is observed for Li_1.5_La_1.5_WO_6_ at 0.35 V and the onset of a pseudo-plateau at ∼0.7 V during charging. On the discharging cycles, a pseudo-plateau is observed below 0.9 V and a plateau at ∼0.2 V. The mixed metal Li_1.5_La_1.5_W_0.5_Te_0.5_O_6_ compound was found to have a discharge capacity intermediate between the parent Li_1.5_La_1.5_*M*O_6_ compounds, confirming the redox activity experienced in these double perovskites is due to the W^6+^ cations. Carbon coating of the Li_1.5_La_1.5_WO_6_ particle surface was performed via a sucrose impregnation–carbonisation route in order to improve the performance and cyclability of the Li_1.5_La_1.5_WO_6_ anode material. Carbon coating improves the electronic properties of the electrode composite and can also act as a buffer layer to protect from continuous side-reactions between the active materials surfaces and the electrolyte. The carbon-coating treatment resulted in an increased discharge capacity (Supplementary Fig. 9) with a value above 200 mAh g^−1^ up to cycle 15, doubling that of the uncoated material’s capacity of ≈100 mAh g^−1^. Retention capacity is also greatly improved with the carbon-coating approach, with an increase from 53 to 85% on cycle 15 and from 41 to 62% at the end of cycle 20.

Assessing the redox activity of the tungsten analogue presented a challenge. XAS measurements of W L_III_-edge (Supplementary Fig. 10) were performed on ex situ cycled samples but assigning oxidation state changes are difficult due to overlapping absorption edges. Only small differences in the relative intensity of the L_III_ split peak were observable making it difficult to attribute these directly to oxidation changes of W. Difficulties in the analysis of W oxidation states by XAS has been previously reported in the literature^41^. Instead, we have used magnetometry to follow the reduction from diamagnetic W^6+^ (5d^0^) to paramagnetic species W^5+^ or W^4+^ during cycling. Measurements were conducted on two samples; a fully discharged sample and a sample charged back to 0.4 V (just above the first oxidation process). Magnetic measurements on the cycled materials point to oxidation state changes on the tungsten ions (Supplementary Fig. 11). For materials discharged to 0.01 V, Curie–Weiss paramagnetism indicative of fully localised unpaired electrons is observed arising from 1.43 μ_B_ per formula unit, corresponding to the formation of 0.8 mol of W^5+^ per formula unit. The observed Weiss constant of −120(2) K indicates strong antiferromagnetic coupling between neighbouring magnetic centres. In conjunction with the observed moment this shows that around 0.8 W^5+^ cations per formula unit are interacting strongly, indicating short superexchange distance between these paramagnetic cations. This implies the W^5+^ are evenly distributed throughout the perovskite phase that has a composition of Li_2.3_La_1.5_WO_6_. From galvanostatic measurements, we expect to observe W^4+^ at this voltage, suggesting that not all of the observed reduction leads to formation of localised, unpaired electrons. Instead, the excess 1.2 electrons per formula unit must lead to species that make no significant contribution to the Curie–Weiss behaviour. Delocalisation of some electrons leading to Pauli paramagnetism or a conversion reaction to a diamagnetic species would both be magnetically undetectable in the presence of the dominant signal from the W^5+^. Cycling the material back to 0.4 V, beyond the first oxidation process, revealed a change in the magnetism of the cycled material with a large reduction of the paramagnetic moment to 0.84(3) µ_B_ and a weak positive Curie constant of *θ* = +9(1) K. This indicates almost complete re-oxidation to W^6+^.

**Fig. 7:**
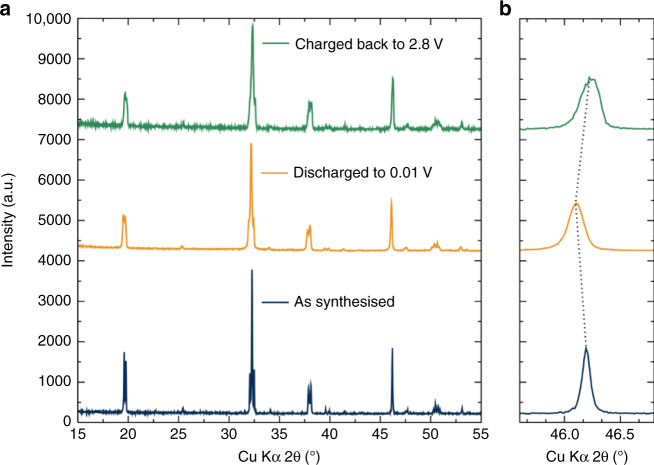
Li_1.5_La_1.5_WO_6_ crystal lattice evolution upon cycling. **a** Ex situ XRD patterns of the Li_1.5_La_1.5_WO_6_ material as-synthesised, after fully electrochemical lithiation and after subsequent de-lithiation (20 cycles) (green line). **b** Magnification of the (220) peak evidences the reversible insertion and de-insertion of Li ions to and from the structure. The calculated volume expansion upon full lithiation is 0.2%.

Ex situ PXRD of fully discharged material revealed that the perovskite structure of Li_1.5_La_1.5_WO_6_ is retained upon lithiation with a small displacement of the diffraction peaks towards higher d-spacing values, which is then reversed when the material is charged back to 2.8 V (Fig. 7). The absence of other phases in these diffraction patterns suggests intercalation as the main mechanism for the observed electrochemical activity of this material. Interestingly, the small lattice parameter change indicates only a small volume increase from 245.016(3) Å before cycling to 245.50(2) Å post-lithiation. This 0.2% change is remarkably low compared to electrodes such as LiFe_0.8_Mn_0.2_SO_4_F, which shows a volume change of 0.6% between the lithiated and delithiated phases^42,43^, or LiFePO_4_ where larger volume changes of 6.6% are noted^44^. The titanate anode material Li_4_Ti_5_O_12_ also possesses a small volume expansion upon cycling (∼0.4%), and intercalates three Li^+^ per Li_4_Ti_5_O_12_ unit giving a similar volumetric capacity to the 2e^−^ reversible cycling we observe per Li_1.5_La_1.5_*W*O_6_^45^. This indicates that Li_1.5_La_1.5_WO_6_ represents a promising anode material for Li-ion batteries, with very low (de)lithiation voltages and extremely low volume expansion upon cycling.

**Fig. 8:**
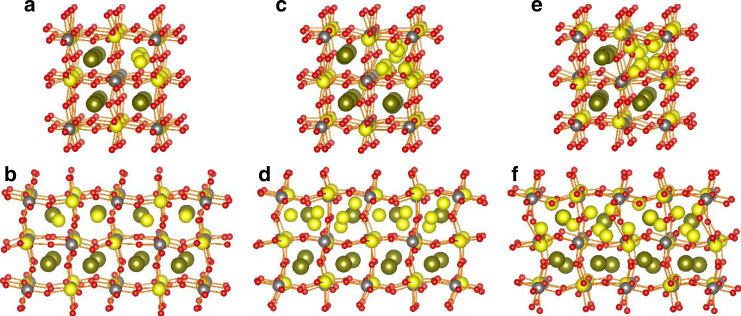
Li_1.5_La_1.5_WO_6_ crystal structure evolution upon Li insertion. **a**, **b** Orthogonal views of the simulated structure of the Li_1.5_La_1.5_WO_6_ crystal structure (representing Li, W, La and O by yellow, grey, green and red spheres, respectively) illustrating preferential ordering of Li^+^ cations into chains of face-sharing *A* sites. **c**, **d** Insertion of additional lithium up to Li_2.5_La_1.5_WO_6_ generates a cluster of three LiO_4_ units within these *A* sites, with a Li···Li cations separation distance of ≥2.44 Å. **e**, **f** Further reductive intercalation to Li_3.0_La_1.5_WO_6_ leads to accommodation of four Li^+^ cations in the *A* site with shortened Li···Li distances and reduced screening as the oxide coordination around these Li^+^ cations become increasingly distorted. A progressive distortion of the perovskite framework is observed with increasing insertion of Li^+^ cations as it adjusts to accommodate the high concentration of Li^+^ in a single *A* site.

Analysis of diffraction data necessarily delivers a crystallographic model that is averaged over many unit cells and requires consideration of local atomic configurations. The disordered distribution of La^3+^ and Li^+^ identified in the Rietveld analysis, and the predicted sites for Li^+^ intercalation were probed using computer simulation. Various cation-ordering schemes were computationally tested using a combination of site occupancy disorder^46^ and molecular dynamics. These employed a supercell containing Li_6_La_6_W_4_O_24_ atoms with no symmetry constraints apart from periodic boundary conditions of *ca*. 7.9 Å in *x, y* and *z* directions. In Li_1.5_La_1.5_WO_6_, Li^+^ occupies a quarter of the *A* sites, and the simulations indicate that there is a small energy stabilisation (<10 kJ mol^−1^ per Li_1.5_La_1.5_WO_6_) for the lithium occupancy to be in adjacent *A* sites as shown in Fig. 8. With such a small difference in energy, we would not expect these weakly favoured local correlations in *A*-site occupancies to lead to a lowering of crystallographic symmetry from the observed monoclinic structure. These observations are in agreement with results for similar systems^19^.

The effect of electrochemically inserting additional lithium was modelled by placing lithium in the largest voids in the structure and minimising the energy over the whole supercell (see Supplementary Note 1 for further details). The resultant structures show that reductive insertion of lithium is most readily achieved by incorporation of additional lithium into the large *A* site interstices that already contain Li^+^, with displacement of the Li^+^ cations away from the centre of the hole to give lower coordination numbers and minimising the electrostatic repulsion arising from Li···Li interactions. The structure for Li_2.5_La_1.5_WO_6_ contains three Li^+^ cations occupying a single *A* site forming tetrahedral LiO_4_ units and maintaining a minimum Li···Li separation of at least 2.44 Å. This stoichiometry corresponds to reduction to W^5+^ and is in agreement with that indicated by the magnetometry measurements. Simulations can push the Li^+^ content towards a limit of Li_3.0_La_1.5_WO_6_ giving four Li^+^ cations in the *A* site. This configuration involves somewhat shorter Li···Li distances, down to 2.29 Å, and considerable distortion of the LiO_6_ and WO_6_ units. Simulations on Li_1.5_La_1.5_TeO_6_ show that, as expected, the 4d^10^ configuration of Te^6+^ presents a barrier to reductive intercalation. Interestingly, Bader charge analysis of the oxidation states of tungsten in the Li_1.5_La_1.5_WO_6_ systems shows a defined stepwise change from Li_1.50_La_1.5_WO_6_ (W^6+^, d^0^) to Li_2.50_La_1.5_WO_6_ (W^5+^, d^1^) to Li_3.00_La_1.5_WO_6_ (W^4+^ and W^5+^ in 50:50 ratio, suggesting mixed d^1^ and d^2^ configurations) (Supplementary Fig. 12). Above Li_3.00_La_1.5_WO_6_ the structure becomes heavily distorted and the electronic structure no longer follows a simple trend. Analogous calculations on the Li_1.5_La_1.5_TeO_6_ structure (Supplementary Fig. 13 and Supplementary Table 7) have revealed large intercalation voltages for Li^+^ intercalation together with reluctance of Te^6+^ to form Te^5+^, suggesting redox cycling of the Te analogue to be unlikely, as experimentally observed. Density of state analysis (Supplementary Fig. 14) also confirms the stability of this material against oxidation, with a large band gap of *ca*. 5 eV in the case of the Li_1.5_La_1.5_TeO_6_ material, indicating high electrochemical stability as solid-state electrolyte.

In order to account for the observed two-electron transfer during cycling, we must consider alternative processes that may contribute to this increased capacity, such as conversion reaction to Li_2_O^47^. From diffraction patterns of the cycled Li_1.5_La_1.5_WO_6_ materials, there is no evidence of crystalline conversion products and scanning electron microscopy (SEM) images of post-cycled material do not suggest significant degradation of the electrode (Supplementary Fig. 15). To shed more light onto the possible mechanism responsible for the additional capacity, we further examined the mixed metal Li_1.5_La_1.5_W_0.5_Te_0.5_O_6_ phase. Interestingly, Li_1.5_La_1.5_W_0.5_Te_0.5_O_6_ does not show a clear low redox couple in contrast to the well-defined peaks observed for Li_1.5_La_1.5_WO_6_. Furthermore, this partial substitution of W^6+^ by Te^6+^ resulted in a reduction of the material specific capacity greater than that corresponding to decreasing concentration of redox active W^6+^. Specifically, the observed capacity on the third cycle of *ca*. 120 mAh g^−1^ in Li_1.5_La_1.5_WO_6_ decreased to *ca*. 50 mAh g^−1^ in Li_1.5_La_1.5_W_0.5_Te_0.5_O_6_, and considering that the carbon black in the redox-inactive Li_1.5_La_1.5_TeO_6_ provides near 25 mAh g^−1^ (Fig. 6a), the reduction of the capacity when replacing half of W^6+^ by Te^6+^ in Li_1.5_La_1.5_W_0.5_Te_0.5_O_6_ is almost fourfold (from ∼95 to 25 mAh g^−1^). In addition to this capacity loss, the long-range crystalline structure of Li_1.5_La_1.5_W_0.5_Te_0.5_O_6_ is largely lost during the first battery discharge (Supplementary Fig. 16) suggesting a conversion-type process may be occurring for this material when reduced. The higher entropy of the resulting Li_1.5_La_1.5_W_0.5_Te_0.5_O_6_ solid-solution phase could be the driving force underpinning the total macroscopic conversion, similarly reported for high entropy oxide materials in energy storage applications^48,49^. The CV data of the Li_1.5_La_1.5_WO_6_ parent compound also show a lower reversibility of the higher voltage redox couple (around 1 V), an observation more commonly found in conversion processes^50–52^. These observations, combined with the results of magnetic measurements and structural simulations, suggest that the low-voltage redox processes observed for the pure tungsten material are a result of intercalation, while the behaviour at higher voltages may involve conversion processes, reminiscent of previous reports for other W^6+^ oxides^53–56^. Similar combined mechanisms have been observed in LiVO_3_, which displays both intercalation and conversion behaviour below 2 V, involving formation of Li_2.5_VO_3_ and conversion into metallic vanadium and Li_2_O^57^. Other bimetallic vanadates have been reported to undergo an initial conversion process within the particles’ surfaces followed by a intercalation process with subsequent Li^+^ insertion^58–60^. Furthermore, unlike the Li_1.5_La_1.5_TeO_6_ material, the Li_1.5_La_1.5_WO_6_ material presents submicron-sized particles on the bulk surface, which could be more prone to conversion reactions (Supplementary Fig. 17).

A key motivation for employing solid electrolytes in all-solid-state batteries is their potential to safely facilitate metallic lithium as an anode material^1,61^. To study the redox stability of Li_1.5_La_1.5_TeO_6_ against lithium metal, an asymmetric cell was assembled using Li metal as a reference electrode and plasma deposited gold as a counter electrode. CV measurements at 80 °C reveal the ability of Li_1.5_La_1.5_TeO_6_ to plate and strip lithium metal with no observable overpotential (Supplementary Fig. 18). Several peaks are observed in the reduction sweep of the first cycle, indicating the possible formation of an interfacial layer^62^. Subsequent cycles reveal that this interfacial layer bestows stability, with no further surface reactions noted. Interestingly, Li_1.5_La_1.5_TeO_6_ displays good stability beyond 5 V making it a candidate for use in high-voltage cells. It should be noted that this is the first work on this novel material and that further optimisation, similar to that experienced by other solid-electrolyte systems (e.g., though microstructure optimisation for better Li wetting, protective layers to avoid reactions with electrodes)^63,64^, is expected.

**Fig. 9:**
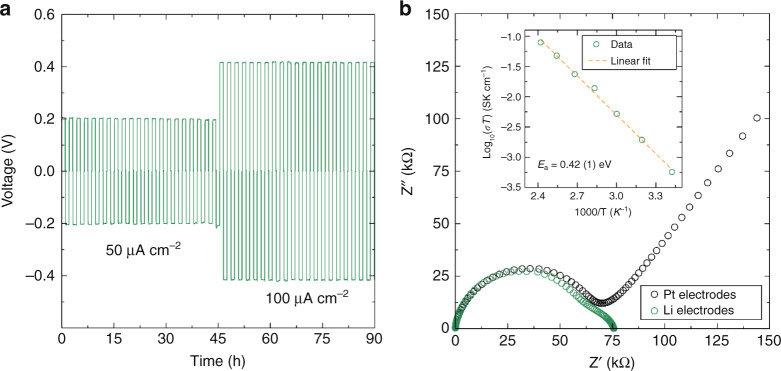
Li_1.5_La_1.5_TeO_6_ stability against Li metal and transport properties. **a** Polarisation test of a symmetric Li|Li_1.5_La_1.5_TeO_6_|Li cell. The applied current densities were 50 and 100 μA cm^−2^ at 80 °C. **b** EIS measurement at 19 °C for a Li|Li_1.5_La_1.5_TeO_6_|Li and a Pt|Li_1.5_La_1.5_TeO_6_|Pt symmetric cells. Inset shows the Arrhenius plot of conductivity measurements at different temperatures using Pt blocking electrodes.

To optimise the ionic conductivity of the Li_1.5_La_1.5_TeO_6_ material as a solid-state electrolyte, highly dense pellets were obtained by SPS. The relative density of the SPS pelleted material was greatly increased from *ca.* 76 to 98.1(1)%. Polarisation tests (Fig. 9a) indicate excellent compatibility and stability between Li metal and Li_1.5_La_1.5_TeO_6_ during plating and stripping with Li electrodes. Impedance analysis of the Li_1.5_La_1.5_TeO_6_ symmetric cells (Fig. 9b) reveals differences in the spectra observed for Li electrodes compared to Pt blocking electrodes. The latter contain a low frequency tail; this contrasts with the second semicircle observed when using Li electrodes, indicating the macroscopic mobility of Li^+^. The small second semicircle observed when using Li electrodes is indicative of a low charge transfer resistance at the Li/Li_1.5_La_1.5_TeO_6_ interface. Improvements in ionic conductivity are observed for the SPS treated Li_1.5_La_1.5_TeO_6_ with a value of 0.12 mS cm^−1^ at 124 °C, doubling that of the cold pressed material. The activation energy for Li^+^ diffusion is also greatly reduced to 0.42(1) eV (Fig. 9b inset). This improvement in transport properties demonstrates the key role that pellet microstructure engineering has on the macroscopic conductivity measured by conventional electrochemical techniques, encouraging dedicated work to optimise this performance further. Subsequent improvement of conduction properties in novel oxide materials following their original report in the literature is often observed. For instance, benchmark Li-rich garnets oxides were originally reported to have ionic conductivities on the order of 10^−6^ S cm^−1^ with activation energies in the 0.4–0.5 eV range, comparable with our novel Li_1.5_La_1.5_TeO_6_ double perovskite, and improvements in the last decade have seen these values rise to above 10^−3^ S cm^−1^ with activation energies below 0.2 eV^65–67^. The low local Li^+^ activation energy below 0.2 eV and similar Li^+^ diffusion coefficient obtained by µ^+^SR here for the Li_1.5_La_1.5_TeO_6_ material is comparable to that of the LLZO benchmark garnet electrolyte probed by the same technique, where again pellet microstructure greatly impacts the macroscopic transport properties^21^. This reinforces the scope for future improvements on the macroscopic transport properties of the Li_1.5_La_1.5_TeO_6_ double perovskite reported here.

**Fig. 10:**
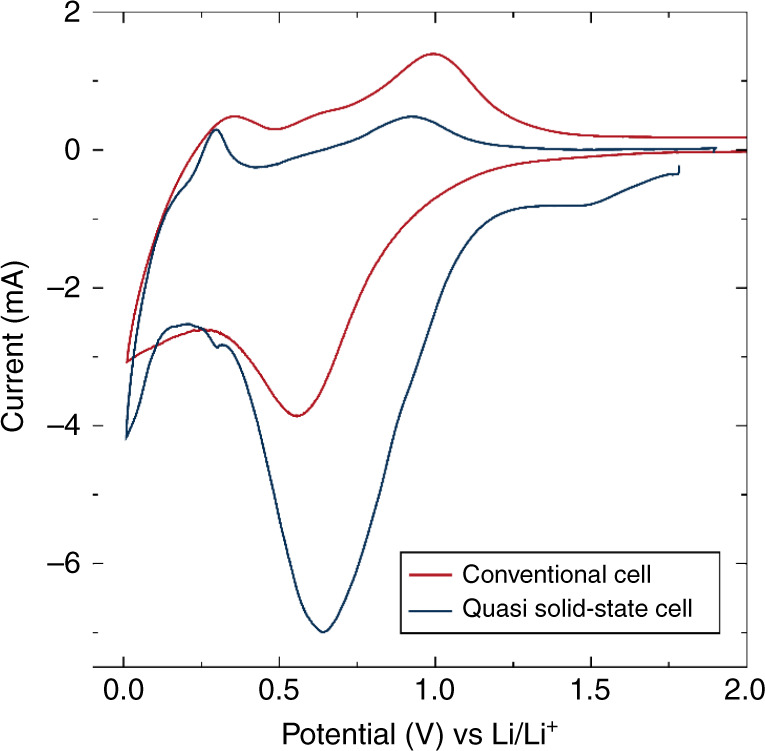
Li_1.5_La_1.5_WO_6_–Li_1.5_La_1.5_TeO_6_ compatibility in a single quasi all-solid-state battery. CV of a Li half-cell formed by Li_1.5_La_1.5_WO_6_:CB:PTFE (90:5:5%_wt_) as active material and a Li_1.5_La_1.5_TeO_6_:LiTFSI:Py_14_TFSI (80:1:19%_wt_) hybrid electrolyte tested against Li metal at 0.05 mV s^−1^ in the voltage range 0.01–1.90 V at 80 °C. For comparison, a CV curve of the Li_1.5_La_1.5_WO_6_ perovskite material in a conventional cell with 1 M LiPF_6_ in EC:DMC (1:1%_vol_) liquid electrolyte cells has been included.

To evaluate the efficacy of the approach of crystal structure matching across the electrode–electrolyte interface, we tested the compatibility of the Li_1.5_La_1.5_WO_6_ low-voltage negative electrode with the Li_1.5_La_1.5_TeO_6_ solid-state electrolyte in a quasi-solid-state battery. This was carried out using a Li-metal half-cell comprising by the Li_1.5_La_1.5_WO_6_ material as the electrode material and a hybrid electrolyte formulation Li_1.5_La_1.5_TeO_6_:LiTFSI:Py_14_TFSI (80:1:19%_wt_) [Py_14_TFSI = 1-butyl-1-methylpyrrolidinium bis(trifluoromethylsulfonyl)imide] without the need for a separator or liquid electrolyte. The presence of the Py_14_TFSI ionic liquid affords better wettability between the electrode and solid-electrolyte phases as well as lowering the resistance of Li^+^ diffusion through the Li_1.5_La_1.5_TeO_6_ solid-electrolyte under the conditions employed. CV analyses at 80 °C (Fig. 10) reveal the clear redox response of Li_1.5_La_1.5_WO_6_, reminiscent of that observed for the conventional liquid electrolyte cell. The intense reduction peak at ∼0.63 V is most likely due to the irreversible formation of SEI at the carbon black surface from the P_14_TFSI ionic liquid, in agreement with that also observed for the conventional liquid electrolyte cell. The low-voltage redox peaks appear more defined and sharper when the Li_1.5_La_1.5_TeO_6_ electrolyte is employed, indicating improved kinetics for Li^+^ transference and insertion. The additional irreversibility observed during the first cycle of the Li_1.5_La_1.5_TeO_6_ CV in the Li|Li_1.5_La_1.5_TeO|Au cell (Supplementary Fig. 18) could be arising from additional interphase formation or initial decomposition at the Li_1.5_La_1.5_TeO_6_ interface, as observed in other solid-state electrolyte systems^68–70^.

## Discussion

We have demonstrated that the tungsten and tellurium analogues of the Li-rich double perovskite family, Li_1.5_La_1.5_*M*O_6_, are excellent candidate electrode and solid-electrolyte materials, respectively, for Li-ion batteries. The presence of Li ions in both *A* and *B* sites within the double perovskite framework enables Li-ion motion, and tailoring of the *B*-site cation directs the functionality towards a low-voltage negative electrode (Li_1.5_La_1.5_WO_6_) or a solid-state electrolyte (Li_1.5_La_1.5_TeO_6_). A detailed investigation into the redox mechanism of these materials unveils W^6+^ as the redox active species during Li insertion, whilst Te^6+^ confers a high redox stability, beyond 5 V, onto the perovskite crystal structure enabling its use as a safe alternative solid-state electrolyte. The promising diffusion properties of this new solid-electrolyte family presents an exciting opportunity for further performance optimisation through microstructure engineering and chemical composition exploration, reminiscent of previous oxide systems such as the Li-rich garnets or NASICON phases^6,34,71,72^. Interestingly, the combination of both Li-rich double perovskites into a single hybrid solid-state cell retains the electrode functionality and paves the way for tailored isostructural design of solid-state battery components whereby interface compatibility can be finely tuned at the unit cell level.

## Methods

All reagent-grade chemicals employed for the synthesis of the Li-rich double perovskites were purchased from the following suppliers and used without further purification unless otherwise noted: LiOH·H_2_O (98%) and La_2_O_3_ (99%) from Sigma Aldrich, WO_3_ (99.95%) and TeO_2_ (99.978%) were purchased from Alfa Aesar.

For the microwave-assisted synthesis of the Li_1.5_La_1.5_WO_6_, Li_1.5_La_1.5_TeO_6_ and Li_1.5_La_1.5_W_0.5_Te_0.5_O_6_ double perovskites, stoichiometric amounts of La_2_O_3_ (previously dried at 900 °C for 24 h), WO_3_ and/or TeO_2_, and a 10% excess of LiOH·H_2_O (^7^LiOH·H_2_O for NPD studies) were ball milled for 30 min at a vibrational frequency of 20 Hz in a stainless-steel jar. Subsequently, the fine powder was pelleted under uniaxial load of 3 t. The pelleted material was heated at 700 °C for 6 h in a 2.45 GHz CEM Phoenix hybrid microwave furnace for the decomposition of the precursor materials. Subsequently, the material was reground and pelleted for a second heat treatment carried out in air at 900 °C for 6 h in the same microwave furnace. The last treatment consisted in 1 h at 1000 °C of the repelleted material in the same hybrid microwave furnace. In every calcination, the heating rate was held at 2 °C min^−1^ to reduce lithium evaporation.

Powder XRD was employed for the assessment of the purity and study of the crystal parameters for compounds prepared. A PANalytical X’Pert PRO Diffractometer was used for this purpose using Cu-Kα radiation in the 2θ range 15–130° with a nominal scan rate of 800 s per step and a step size of 0.016° at room temperature.

NPD patterns used for Rietveld refinements were collected in Bank 3 (2θ = 35.26°) and Bank 5 (2θ = 91.34°) at the GEM instrument for the Li_1.5_La_1.5_WO_6_ material, and Bank 3 (2θ = 51.99°) and Bank 5 (2θ = 146.94°) at the Polaris instrument for the Li_1.5_La_1.5_TeO_6_ analogue at the ISIS pulsed neutron and muon source at the Rutherford Appleton Laboratory, UK. The data were collected over a time-of-flight region 0.25–23 ms (Polaris) and 0.9–21.5 ms (GEM). The sample, *ca*. 1–3 g, was placed in an 11-mm-diameter cylindrical vanadium can and loaded into the beamline. The data were collected at room temperature. The broad incident pulsed neutron flux was narrowed in a 100 K methane moderator giving a peak flux at *λ* = 2 Å, prior to sample scattering^73,74^. Rietveld refinements against XRD and NPD data were performed with the Generalized Structure Analysis System (GSAS)^75^, along with the graphical user interface EXPGUI^76^, by means of a least square approach.

In order to analyse the size and morphology of the synthesised particles, SEM images were acquired with a Carl Zeiss Sigma microscope. All samples were ground, and a tiny amount of the fine powder was deposited over a carbon-taped sample holder. Subsequently the sample was Au-coated and ready for analysis.

EDX spectra were recorded using an Oxford Instruments Energy 250 energy-dispersive spectrometer system. Copper tape was employed as a standard for calibration and the voltage of the incident beam was 25 keV.

Induced coupled plasma-mass spectroscopy (ICP-MS) analyses were performed on an Agilent 7700 ICP-MS instrument. Approximately 6 mg of sample was dissolved in 50 mL of 2% HNO_3_ solution in deionized water and immersed into an ultrasonic bath for 5 min prior to the measurements.

EIS AC measurements with Pt electrode were performed on a Solartron 1260 Impedance Analyzer in the frequency range of 1–10^6^ Hz and a temperature range between RT and 400 °C in 50 °C steps using as-synthesised pelleted materials. In order to enhance the connection between the pellet and the electrodes, a suspension of 0.5–5 µm platinum particles in *n*-butyl acetate was prepared and a few drops of this suspension were deposited on both surfaces of the pelletized material. The electrodes consisted of square pieces of 0.025-mm-thickness platinum foil, connected through 0.127-mm-diameter platinum wire to the device.

CV and galvanostatic cycling measurements were conducted in a BioLogic VSP3 potentiostat using two electrode Swagelok type cells. The electrode material was formed by a mixture of the double perovskite material (90%), electronically conductive carbon black Ketjenblack EC600JD (AkzoNobel) (5%) and PTFE (polytetrafluoroethylene) (Fischer Scientific) (5%) as binder to the electrode mixture. A Whatman glass microfibre filter (GF/D grade) was used as a separator with 1 M LiPF_6_ in ethylene carbonate and dimethyl carbonate 1:1 v/v (Solvionic) as the electrolyte and a 10-mm-diameter circular piece of Li metal of 0.75 mm thickness (Sigma Aldrich) as the reference and counter electrode. C-coated Li_1.5_La_1.5_WO_6_ (targeting 10%_wt_ and resulting in a 6%_wt_ real content as calculated from EA) was produced by sucrose route previously employed in our group^77^. In brief, Li_1.5_La_1.5_WO_6_ as-synthesised material was mixed with sucrose in a 50:50 (%_vol_) ethanol:water solution. The resulting suspension was sonicated for 30 min and subsequently heated until the solvent evaporated. The mixture was then dried under vacuum at 80 °C for 12 h before carbonisation in a tube furnace under flowing Ar gas for 3 h at 700 °C. For the Li_1.5_La_1.5_TeO_6_ perovskite material, an asymmetrical cell composed of pelletised Li_1.5_La_1.5_TeO_6_ material sandwiched between Li metal as reference and counter electrode and sputtered gold as working electrode was mounted on a Swagelok cell at 80 °C for CV analyses on the same VSP3 instrument at a voltage scanning speed of 0.05 mV s^−1^. In the case of the polarisation test and EIS of the symmetrical Li_1.5_La_1.5_TeO_6_ cell with Li-metal electrodes, the same set-up was employed by replacing the Au layer with a Li-metal electrode.

SPS experiments were performed in an FCT HP D 25 SPS furnace. Powder samples were loaded into a cylindrical graphite die with a 16-mm-inner diameter, lined with thin graphite foil. Rapid heating was controlled by a thermocouple inserted within the die for the duration of the experiment. The set-up was held under constant uniaxial pressure of 50 MPa whilst DC current pulses were used to heat the sample at a constant rate of 50 °C/min via Joule heating between particles. The target temperature was 1090 °C with a dwell time of 5 min once this was reached. After the experiment, the sample was allowed to cool naturally. The material was then removed from the die, the surface graphite removed, and polished using sandpaper of up to 2500 grit to obtain smooth pellets of ∼12 mm diameter and 1.5 mm thickness. AC impedance and galvanostatic cycling measurements of SPS samples were performed on a Biologic VSP potentiostat in the frequency range of 1 Hz to 7 MHz with a 50 mV voltage perturbation. Gas deposition was employed to sputter coat electrodes in Pt using a Polaron SC7640 with a sputtering time of 200 s, a current of 20 mA and an Ar pressure of ∼0.02 mbar. The same pellet was used for measurements with lithium electrodes, whereby the pellet was polished to remove the Pt coating and transferred to an Ar-filled glovebox. The pellet was sandwiched between two lithium foils (Sigma Aldrich, 0.38 mm) and assembled within a Swagelok cell. The surface of each lithium foil was scraped using a stainless-steel blade to ensure optimal contact with the pellet.

Density values were obtained through helium gas displacement pycnometry using a Micromeritics AccuPyc II 1340 system.

Raman data were acquired in a Horiba Jobin Yvon LabRAM HR system equipped with a Ventus 532 laser. Spectra were acquired in the 50–4000 cm^−1^ Raman shift range with a 532-nm-wavelength laser with a 100 mW power. The light beam was masked to the appropriated level in order to obtain data intensity in a measurable range.

The XAS data were collected in the B18 beamline at Diamond Source of Light synchrotron. For the data acquisition, a few milligrams (between 10 and 100 mg) of the as-synthesised materials were mixed with cellulose fibre (*ca*. 100 mg) and compacted into a 10-mm-diameter thin pellet and stored in an aluminium plastic bag pocket under Ar inert atmosphere. Samples were mounted into a holder and exposed to the synchrotron X-ray radiation emitted by a bending magnet source which is monochromatised and focused by a vertically collimating Si mirror, a water-cooled Si(111) and Si(311) double crystal monochromator and a focusing double toroidal mirror. The data were collected in the transmission mode using three ionisation chambers mounted in series for simultaneous measurements on the sample and a tungsten foil as reference. Scans of *ca*. 3–5 m were collected over the desired energy range and merging of three consecutive scans was performed to obtain precise data sets. The data were processed and normalised using the Athena software package using edge step normalisation. For fits of the EXAFS data, the Artemis software package was employed. The Artemis software allows one to perform FEFF calculations of the theoretical EXAFS from the crystal data obtained by diffraction methods to obtain the individual scattering pathways, which then can be fit to the experimental EXAFS in order to compared atomic distances in the local and the average structures. Through the incorporated IFEFFIT algorithm, the data were fitted by a least-squares procedure until the best possible fit was achieved^78^.

µ^+^SR studies were performed using the EMU instrument at the ISIS pulsed muon facility^79^. The powdered sample, *ca*. 1.5 g, was packed into a disk of 30 mm diameter and 1.5 mm thickness and sealed in a titanium sample holder where the front window was made of 25-µm-thickness titanium foil. 3.2 MeV spin-polarised positive muons were implanted into the sample and the outcoming positrons were detected by 96 scintillator segments grouped in two circular arrays. The temperature was controlled from 100 K up to a maximum of 600 K by a hot stage attached to a closed cycle refrigerator and the measurements were acquired at three different applied longitudinal magnetic fields (0, 5 and 10 G). A 20 G transverse magnetic field was also applied for the initial asymmetry calibration. The data fits were carried out through the WiMDA data analysis programme^80^.

Magnetic measurements were conducted by using a Quantum Design MPMS-XL SQUID magnetometer to measure *ca*. 20 mg of cycled material sample contained in a gelatine capsule. Data were collected in an applied field after cooling in either the measuring field or in zero applied field and were not corrected for diamagnetism. Susceptibility data were fitted using the Curie–Weiss law in the temperature range 150–290 K.

DFT calculations employed the Vienna Ab initio Simulation Package with the projector augmented wave method^81^ and the PBEsol functional^82^. An additional Dudarev U_eff_ parameter, obtained from the materials project database, was used to afford a more accurate representation of any d-orbital electrons of tungsten^83^ (GGA+U calculations: https://materialsproject.org/wiki/index.php/GGA%2BU_calculations). A 700 eV plane-wave energy cutoff was used and spin polarisation was introduced. A varying Γ-centred Monkhorst–Pack k-point mesh was employed depending on the size of the simulation cell. The Li_6_La_6_W_4_O_24_ system used a converged k-point mesh of 2 × 2 × 2. Lattice vectors were allowed to vary during minimisations and a force tolerance of 0.01 eV Å was applied for convergence. The minimisations are followed by a single point energy calculation with the HSE06 functional at the Γ-point. The use of the hybrid HSE06 functional better accounts for localisation of d electrons and therefore was used for Bader charge and electronic structure analysis. Further details on computational analyses are found in the Supplementary Information.

## Supplementary information

Supplementary Information

Peer Review File

## Data Availability

The data sets generated and analysed during the current study are available from the corresponding author on reasonable request. The data sets generated using the ISIS neutron and muon source are available from ISIS Neutron and Muon Source Data Catalogue using 10.5286/ISIS.E.RB1620255 and 10.5286/ISIS.E.RB1720318 for muon and neutron data sets, respectively.
