# Supplementary material for: Li1.5La1.5MO6 (M = W6+, Te6+) as a new series of lithium-rich double perovskites for all-solid-state lithium-ion batteries
Source: Nat Commun. 2020 Dec 15;11:6392. doi: 10.1038/s41467-020-19815-5 (PMC7738526; doi:10.1038/s41467-020-19815-5)
Supplement: Supplementary file 1 — Supplementary Information [file 41467_2020_19815_MOESM1_ESM.docx]

**Supplementary Information**

**Li_1.5_La_1.5_*M*O_6_ (*M* = W^6+^, Te^6+^) as a new series of lithium-rich double perovskites for all-solid-state lithium-ion batteries**

Marco Amores,^1^ Hany El-Shinawi,^1,2^ Innes McClelland,^1^ Stephen R. Yeandel,^3^ Peter J. Baker,^4^ Ronald I. Smith,^4^ Helen Y. Playford,^4^ Pooja Goddard,^3^ Serena A. Corr^1,5*^ and Edmund J. Cussen^1,5*^

1. *Department of Chemical and Biological Engineering, University of Sheffield, Sheffield S1 3JD, UK*
2. *The Faraday Institution, Harwell Campus, Didcot OX10RA, UK*
3. *Department of Chemistry, Loughborough University, Epinal Way, Loughborough LE11 3TU, UK*
4. *ISIS Pulsed Neutron and Muon Source, STFC Rutherford Appleton Laboratory, Harwell Science and Innovation Campus, Didcot, Oxfordshire OX11 0QX, UK*
5. *Department of Materials Science and Engineering, University of Sheffield, Sheffield S1 3JD, UK*


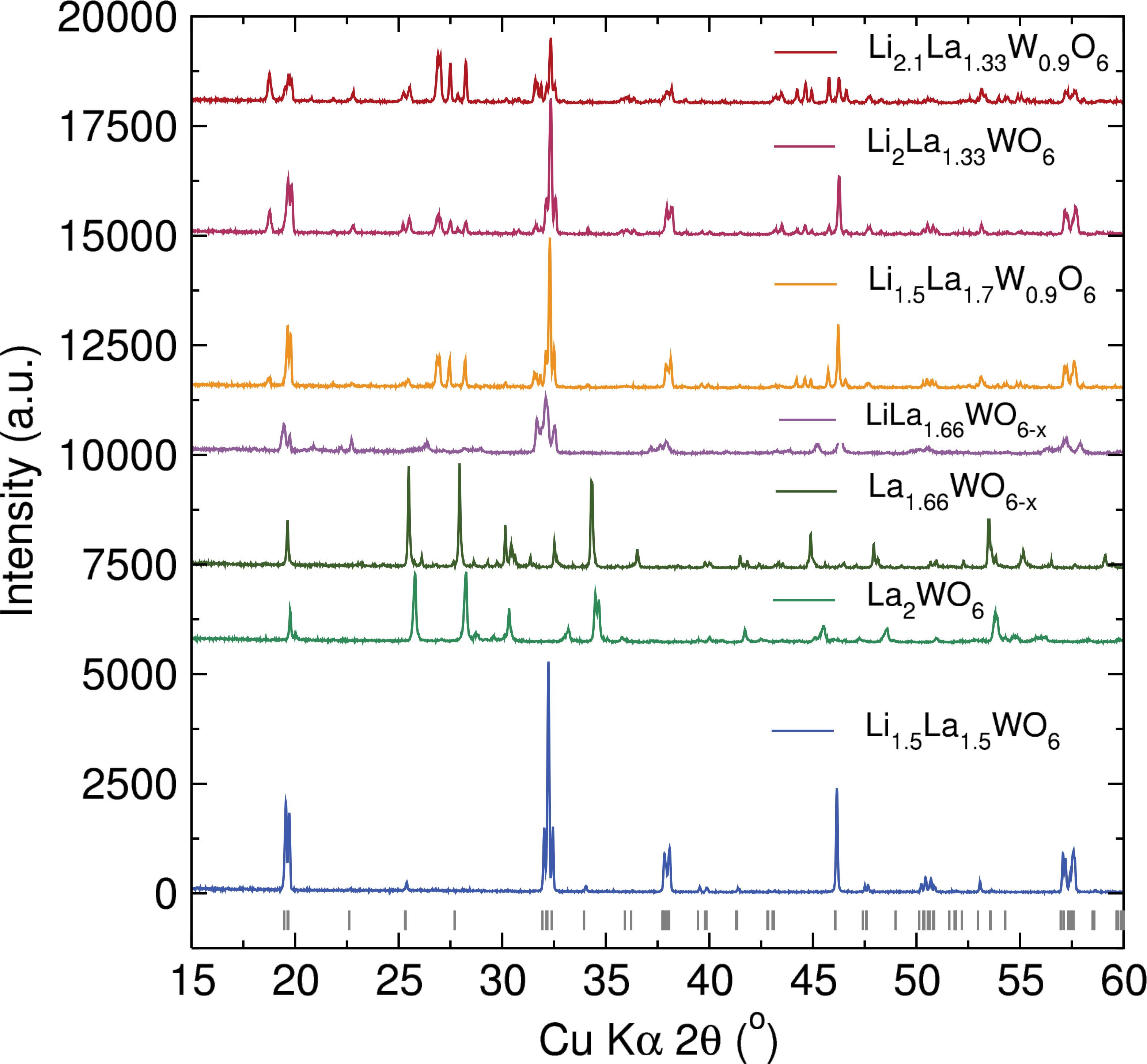


**Supplementary Figure 1:** PXRD data of the compositional scan of different Li:La:W stoichiometries. It is clear that only our employed stoichiometry lead to phase pure material. All samples were subjected to the same heat treatment as the Li_1.5_La_1.5_WO_6_ sample.

**Supplementary Table 1**: Atomic parameters extracted from Rietveld Refinements to PXRD and NPD data for the Li_1.5_La_1.5_WO_6_ fit to the monoclinic (*P*2_1_/*n*) space group. R_wp_ = 4.68, R_p_ = 5.43 and χ^2^ = 3.36. *a* = 5.53537(6) Å, *b* = 5.61275(5) Å, *c* = 7.88629(9) Å, β = 90.105(1)°, V = 245.016(3) Å. Atomic parameters from the refinements where all non-special atomic parameters were refined without restrictions.

| Atom | Site | Frac. | x | y | z | 100 U_iso_ / Å^2^ |
| --- | --- | --- | --- | --- | --- | --- |
| La1 | 4e | 0.717(6) | 0.0082(3) | 0.9613(2) | 0.7488(2) | 0.44(4) |
| Li1 | 2d | 0.99(3) | ^1^/_2_ | 0 | 0 | 0.5(1) |
| Li2 | 4e | 0.20(1) | 0.501(5) | 0.355(5) | 0.224(5) | 2.3(2) |
| O1 | 4e | 1.00(1) | 0.2780(4) | 0.6947(5) | 0.9579(4) | 1.20(9) |
| O2 | 4e | 0.99(1) | 0.1923(4) | 0.2210(5) | 0.9574(4) | 1.11(9) |
| O3 | 4e | 1.00(1) | 0.9168(4) | 0.5150(3) | 0.7634(4) | 1.35(8) |
| W1 | 2c | 0.971(9) | ^1^/_2_ | 0 | ^1^/_2_ | 1.09(6) |

**Supplementary Table 2:** Atomic percentages of La, Te and W obtained by EDX measurements at 25 kV on as-synthesised powders. The atomic ratio has been calculated using W atomic percentage as reference for Li_1.5_La_1. 5_WO_6_ (LLWO) and Li_1.5_La_1. 5_W_0.5_Te_0.5_O_6_ (LLWTeO), and Te for the Li_1.5_La_1. 5_TeO_6_ (LLTeO) material.

| Material | La (atomic %) | Te (atomic %) | W (atomic %) | La:W:Te ratio |
| --- | --- | --- | --- | --- |
| LLWO | 60.0(7) | N/A | 40.0(7) | 1.50:1:0 |
| LLWTeO | 60.6(6) | 19.5(1) | 19.9(5) | 1.52:0.49:0.5 |
| LLTeO | 60.2(4) | 39.8(4) | N/A | 1.51:0:1 |

**Supplementary Table 3:** Atomic ratio of Li and La obtained from ICP-MS analyses. The ratios are normalised to La content. Only Li:La ratio was analysed as W content was not possible to obtain by ICP-MS owing to the precipitation and insolubility of WO_3_ in the HNO_3_ digesting solution.

| Material | Li:La ratio |
| --- | --- |
| LLWO | 1.06(4) |
| LLWTeO | 1.075(3) |
| LLTeO | 1.075(1) |


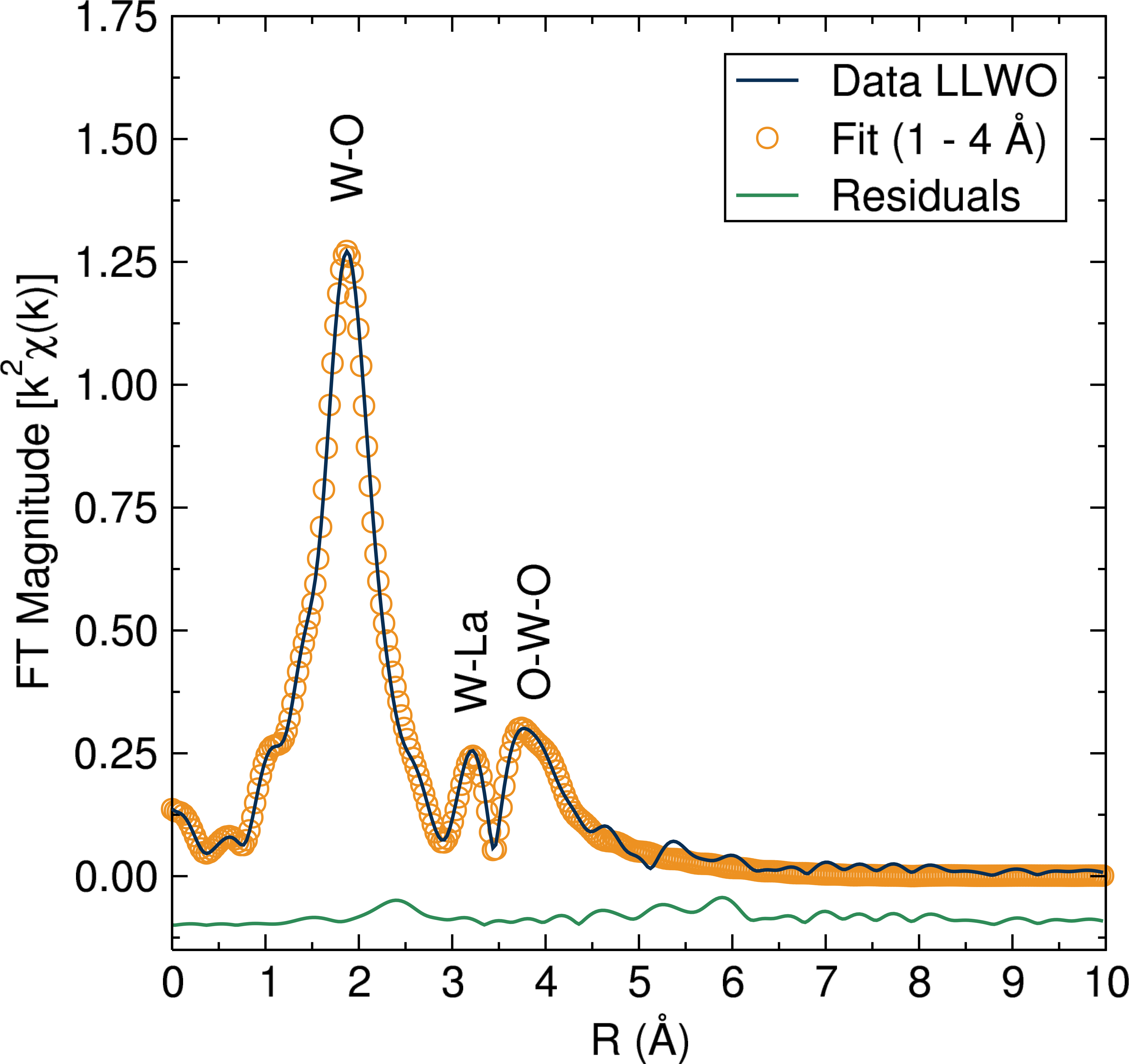


| Atomic Pair | r(EXAFS) (Å) | r(NPD) (Å) |
| --- | --- | --- |
| W-O | 1.872(5) | 1.901(3) |
| W-La | 3.35(1) | 3.368(3) |
| O-W-O | 3.76(2) | 3.801(6) |

**Supplementary Figure 2:** Real part of the Fourier transform for the EXAFS data measured on the W L_III_-edge for the LLWO material. The fit was performed on the 1-4 Å windows using data from the 4.0 - 13.3 Å^-1^ k-range with a final fitting R-factor of 0.0016. The data are represented phase-corrected using the W-O first shell. Effective distances fitted for the main path contributing to the EXAFS spectrum and the distances obtained from PXRD and NPD diffraction data are shown in the bottom table for comparison.

**
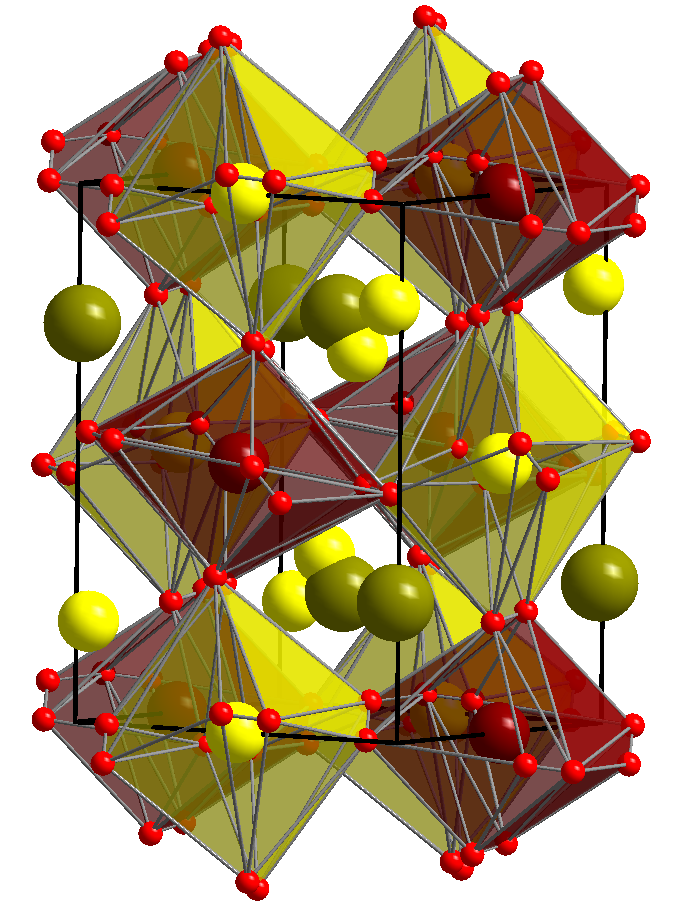
**

**Supplementary Figure 3**: Monoclinically distorted double perovskite structure of Li_1.5_La_1.5_TeO_6_. Red spheres in the octahedra represent Te^6+^ ions, dark green spheres represent La^3+^ ions and Li^+^ ions are represented by yellow spheres. Structure reveals the disordered arrangement of the oxide sublattice.

**Supplementary Table 4** Structural information derived from simultaneous Rietveld refinement against X-ray and neutron diffraction data collected from Li_1.5_La_1.5_TeO_6_ at room temperature.

| Atom | Site | Frac. | x | y | z | 100 U_iso_ / Å^2^ |
| --- | --- | --- | --- | --- | --- | --- |
| La1 | 4e | 0.75 | 0.0113(3) | 0.9987(6) | 0.7404(2) | 0.0086(3) |
| Li(1) | 4e | 0.25 | -0.048(6) | 0.006(8) | 0.811(3) | 0.0039(13) |
| Li2 | 2d | 1 | ½ | 0 | 0 | 0.0039(13) |
| O1 | 4e | 0.356(4) | 0.2268(11) | 0.7947(14) | 0.0141(11) | 0.0021(5) |
| O2 | 4e | 0.356(4) | 0.3526(12) | 0.3112(12) | 0.9501(8) | 0.0021(5) |
| O3 | 4e | 0.356(4) | 0.9762(11) | 0.5762 (15) | 0.7929(8) | 0.0021(5) |
| Te1 | 2c | 1.003(3) | ½ | 0 | ½ | 0.0057(3) |
| O1_spl | 4e | 0.644(4) | 0.2669(6) | 0.7178(7) | 0.9443(6) | 0.0021(5) |
| O2_spl | 4e | 0.644(4) | 0.2378(6) | 0.2528(8) | 0.9584(5) | 0.0021(5) |
| O3_spl | 4e | 0.644(4) | 0.9396(7) | 0.4705(10) | 0.7742(4) | 0.0021(5) |

Space group *P*2_1_/*n*: *a* = 5.57152(14) Å, *b* = 5.60915(18) Å, *c* = 7.9109(2) Å, β = 90.566(2)°, V = 247.216(8) Å^3^.


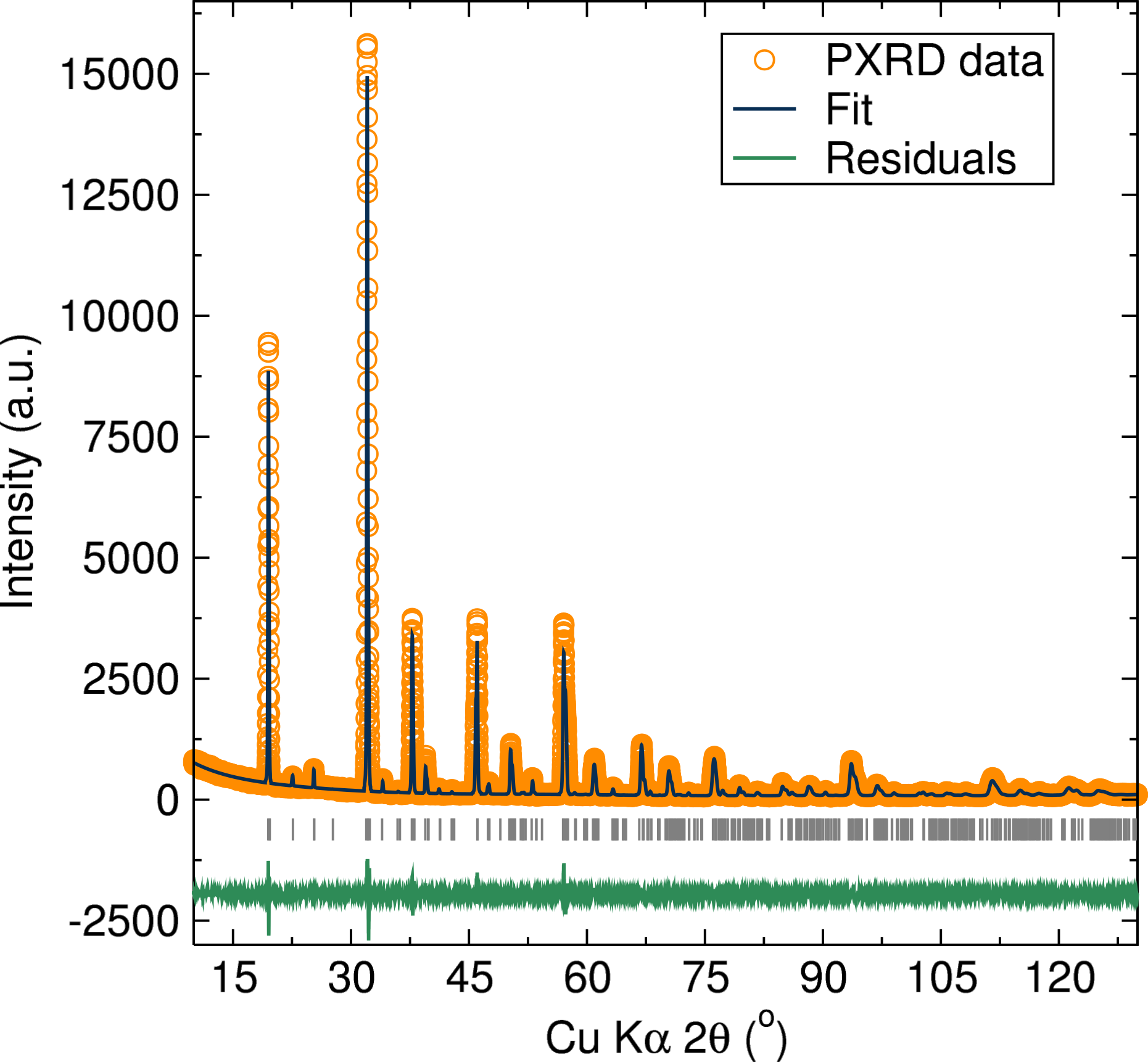


| Atom | Site | Frac. | x | y | z | 100 U_iso_ / Å^2^ |
| --- | --- | --- | --- | --- | --- | --- |
| La1 | 4e | 0.788(2) | 0.0056(5) | 0.9706(2) | 0.7479(2) | 1.75(3) |
| Li1 | 2d | 1 | ^1^/_2_ | 0 | 0 | 0.5 |
| O1 | 4e | 1 | 0.274(2) | 0.700(2) | 0.964(2) | 0.7(1) |
| O2 | 4e | 1 | 0.234(2) | 0.233(2) | 0.955(2) | 0.7(1) |
| O3 | 4e | 1 | 0.889(2) | 0.513(1) | 0.751(1) | 0.7(1) |
| W1 | 2c | 0.5 | ^1^/_2_ | 0 | ^1^/_2_ | 2.02(3) |
| Te1 | 2c | 0.5 | ^1^/_2_ | 0 | ^1^/_2_ | 0.09(2) |

**Supplementary Figure 4**: Rietveld refinements against X-ray powder diffraction data for Li_1.5_La_1. 5_W_0.5_Te_0.5_O_6_ using the monoclinic space group *P*21/*n* with the following cell parameters: *a* = 5.56418(9) Å, *b*= 5.5976(5) Å, *c* = 7.9209(1) Å, β = 90.161(2)°, V = 246.704(9) Å^3^ giving parameters R_wp_ = 9.14, R_p_ = 6.79 and χ^2^ = 2.35. The absence of any secondary impurities, EDX, and ICP analyses, demonstrate that the Li_1.5_La_1.5_W_0.5_Te_0.5_O_6_ intermediate compound possesses similar structural features to the Li_1.5_La_1.5_WO_6_ and Li_1.5_La_1.5_TeO_6_ materials, i.e. there are La^3+^ vacancies on the *A* sites and additional Li ions that cannot be sitting on the fully occupied *B* sites


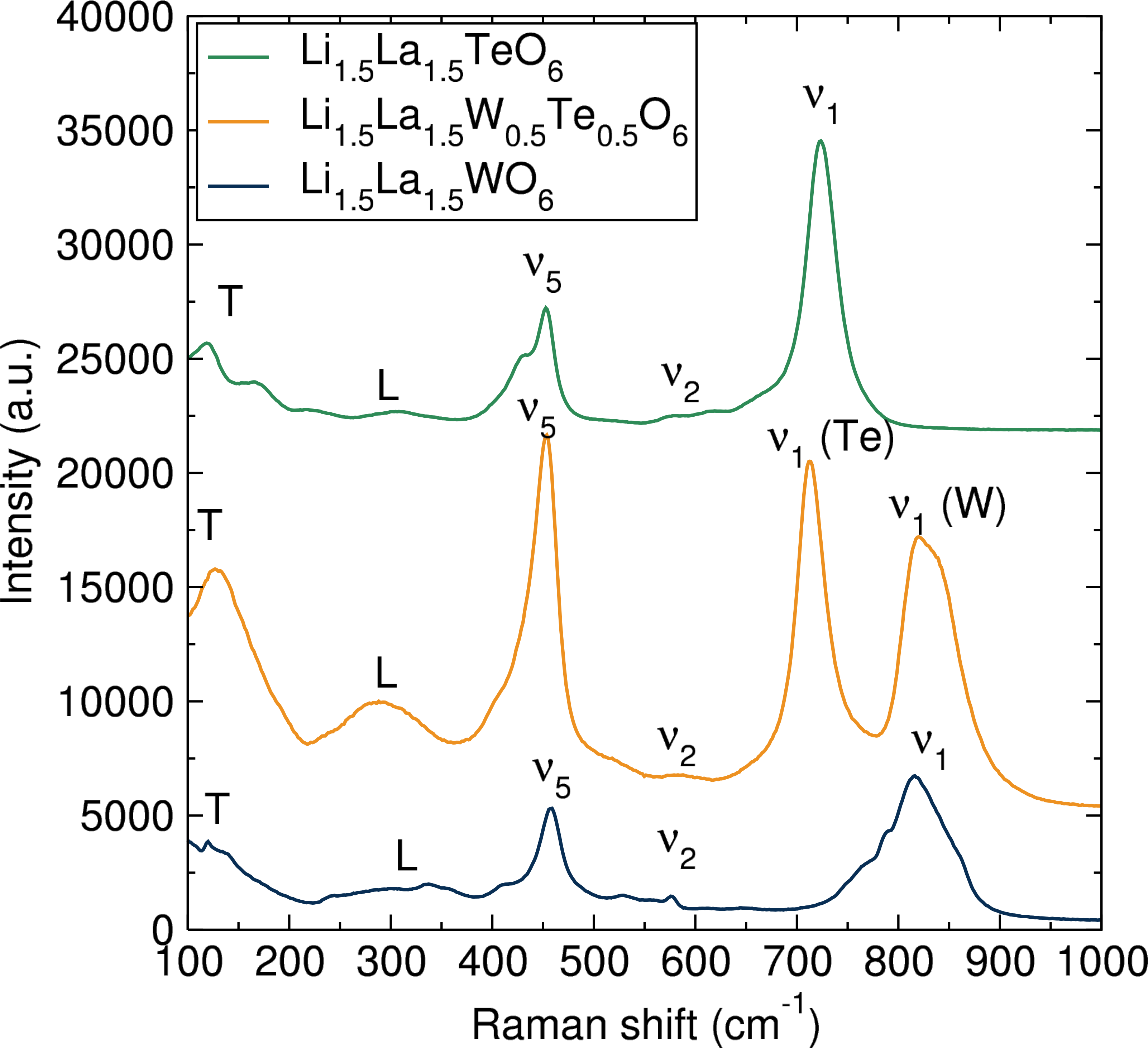


**Supplementary Figure 5:** Raman spectra of the three double perovskites prepared in this work, under excitation of a wavelength λ = 532 nm argon-ion laser. Double perovskites with monoclinic *P*2_1_/*n* group have an irreducible representation Γ = ν_1_(*A*_g_ + *B*_g_) + ν_2_(2*A*_g_ + 2*B*_g_) + ν_5_(3*A*_g_ + 3*B*_g_) + T(3*A*_g_ + 3*B*_g_) + L(3*A*_g_ + 3*B*_g_) for the Raman active modes, where ν, T and L are the vibrational, translational and librational modes, respectively. Vibrational modes are related to the *M*-O stretching modes, the translational modes correspond to the O-M-O bending vibrations and the librational modes are due to the asymmetric MO_6_ octahedral rotation. Librational modes appear at low Raman shift, around 100 to 200 cm^-1^ with multiplicities higher than one due to the asymmetry on the distorted octahedral in the monoclinic structure. These rotational modes cannot be fully resolved due to the proximity to the elastic peak. Translational modes between 200 and 500 cm^-1^, caused by the O-M-O bending vibrations, present a strong split peak at round 450 cm^-1^. The vibrational mode ν_1_ is the most intense peak of the spectra at *ca*. 815 cm^-1^ for the W double perovskite and appears at a lower Raman shift of *ca*. 723 cm^-1^ for the Te analogue, with both modes presented in the mixed metal compound.


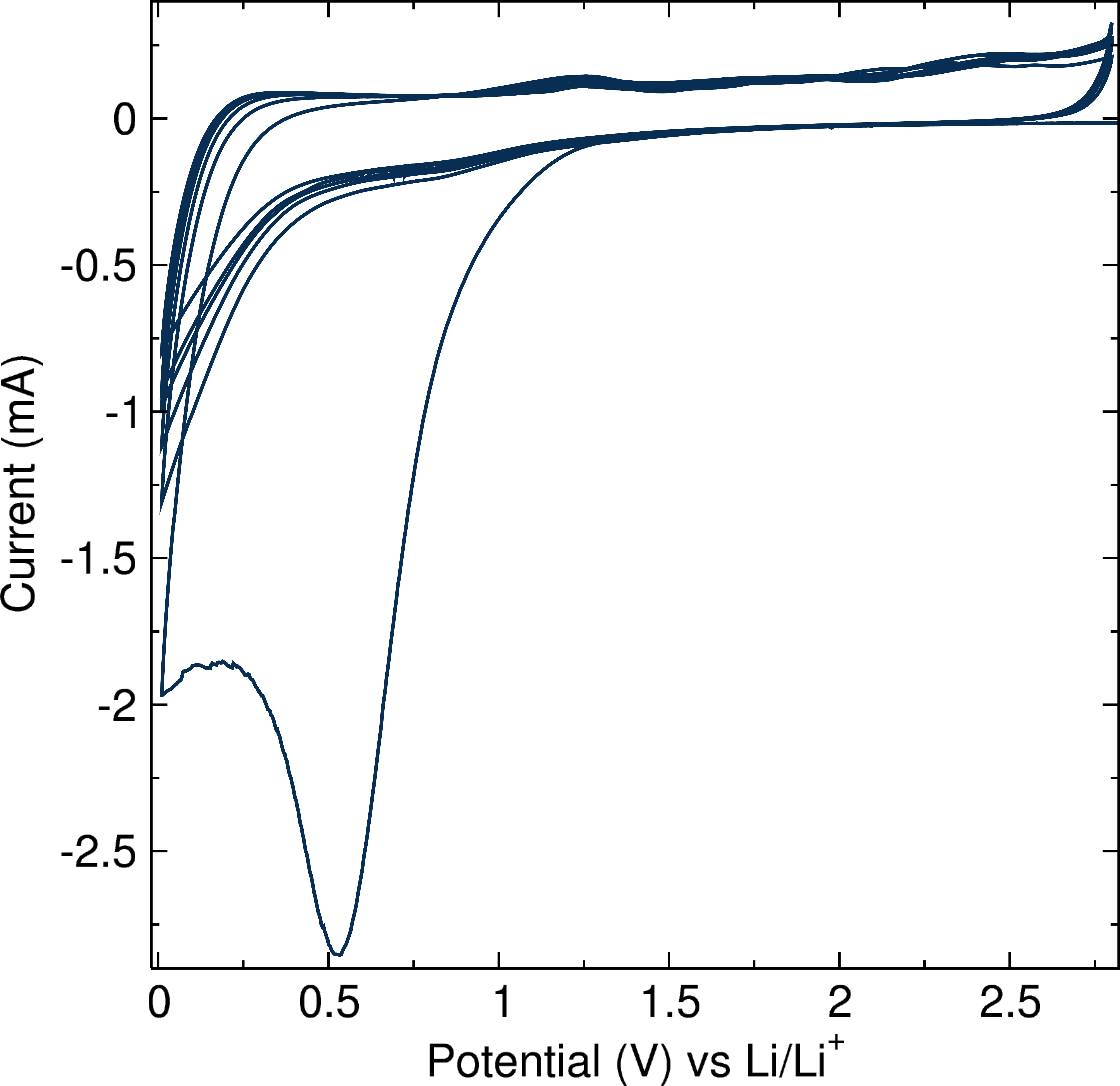


**Supplementary Figure 6:** Cyclic voltammetry data of carbon black and PTFE binder (1:1 by weight) between 0.01 and 2.8 V vs Li. The scan rate was fixed at 0.1 mV s^-1^.


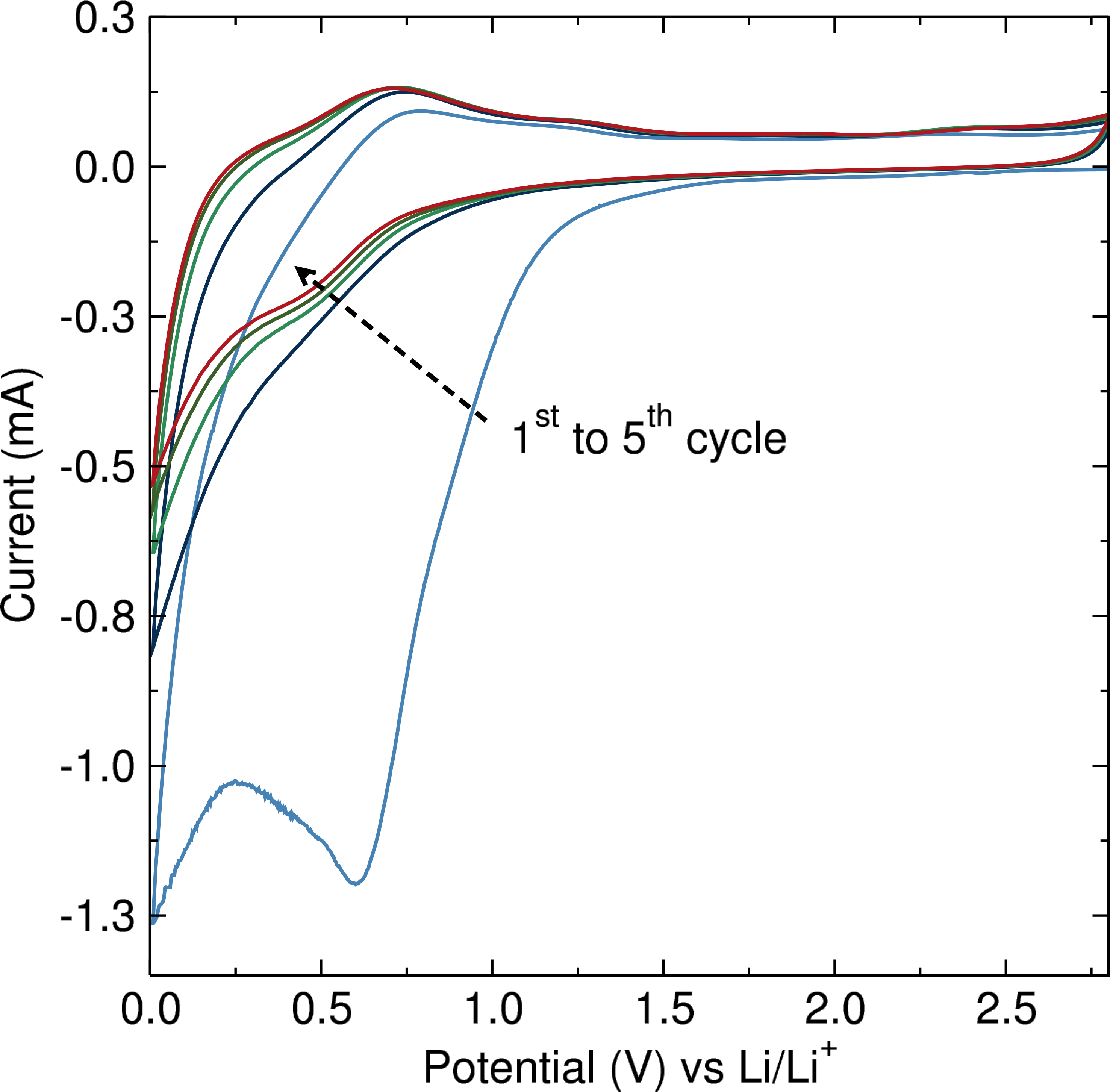


**Supplementary Figure 7:** Cyclic voltammetry data of the Li_1.5_La_1.5_W_0.5_Te_0.5_O_6_ double perovskite material mixed with 5% of carbon black and 5% of PTFE as a binder between 0.01 and 2.8 V vs Li. The scan rate was fixed at 0.1 mV s^-1^.

Supplementary Table 5: Transport properties obtained from electrochemical impedance spectra (using Pt electrodes) showing the total (i.e. inter- plus intra-grain) conductivity for Li_1.5_La_1.5_WO_6_ and Li_1.5_La_1.5_TeO_6_ materials.

| Temp. (K) | Ionic Conductivity LLWO (S cm^-1^) | Ionic Conductivity LLTeO (S cm^-1^) |
| --- | --- | --- |
| 297 | 1.38 × 10^-7^ | 9.28 × 10^-7^ |
| 347 | 1.09 × 10^-6^ | 6.18 × 10^-5^ |
| 397 | 1.11 × 10^-5^ | 5.80 × 10^-5^ |
| 447 | 6.67 × 10^-5^ | 3.74 × 10^-4^ |
| 497 | 3.03 × 10^-4^ | 3.35 × 10^-3^ |
| 547 | 1.31 × 10^-3^ | 1.86 × 10^-2^ |
| 597 | 4.41 × 10^-3^ | 6.82 × 10^-2^ |
| 647 | 1.34 × 10^-2^ | 3.42 × 10^-1^ |
| 697 | 5.02 × 10^-2^ | 6.81 × 10^-1^ |


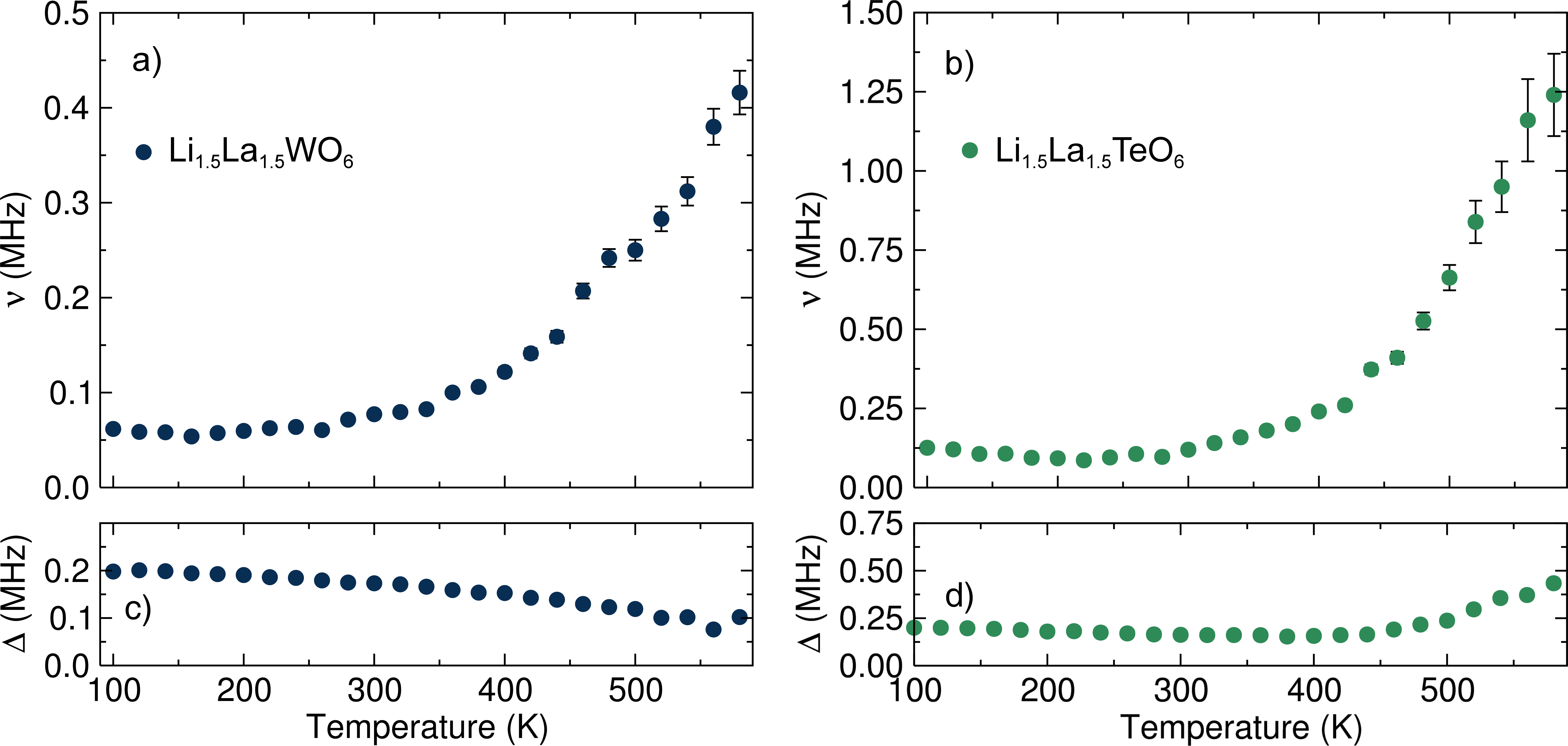


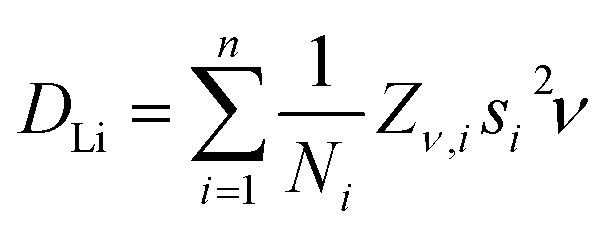
**Supplementary Figure 8:** a) and b) temperature dependence of the muon fluctuation rate (ν) and, c) and d) field width distribution (∆) values obtained for the fits to the Keren function for the W and Te double perovskites materials measured from 100 K to 600 K. Error bars are indicated as rising black lines over the data points. Fits of the muon spin depolarisation data to the Keren function^1^ allowed calculation of evolution of the ν and ∆ parameters at different temperatures. In order to calculate the Li^+^ diffusion coefficients for both materials the structural data obtained from Rietveld refinements to NPD and PXRD data in conjunction with a model for Li^+^ diffusion from the A sites to the two neighbouring B sites and from the B sites to the four adjacent A sites were employed. This model is a simplified version of the diffusion model calculated by DFT for the LLWO material by Rosseinsky and co-workers^2^ where Li^+^ move from A to B sites through an intricate pathway composed by multiple individual hops. The two different Li_A_-Li_B_ distances employed in the calculation were 2.66 and 3.61 Å for the W material and 2.95 and 3.38 Å for the Te material. Diffusion coefficients were calculated by means of the equation:

where *N_i_* is the number of accessible Li sites in the *i*-th path, *Z_ν_*_,_*_i_* is the vacancy fraction of the destination sites, *s_i_* the jump distance between Li sites, and *ν* the calculated fluctuation rate at each temperature.


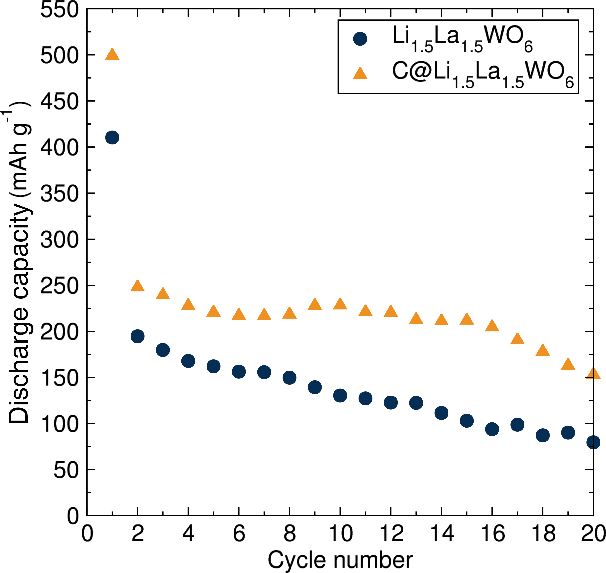


**Supplementary Figure 9:** Discharge capacities observed for Li_1.5_La_1.5_WO_6_ Li-rich double perovskite anode material with (orange triangles) and without carbon coating (blue spheres) cycled at 17 mA g^-1^.


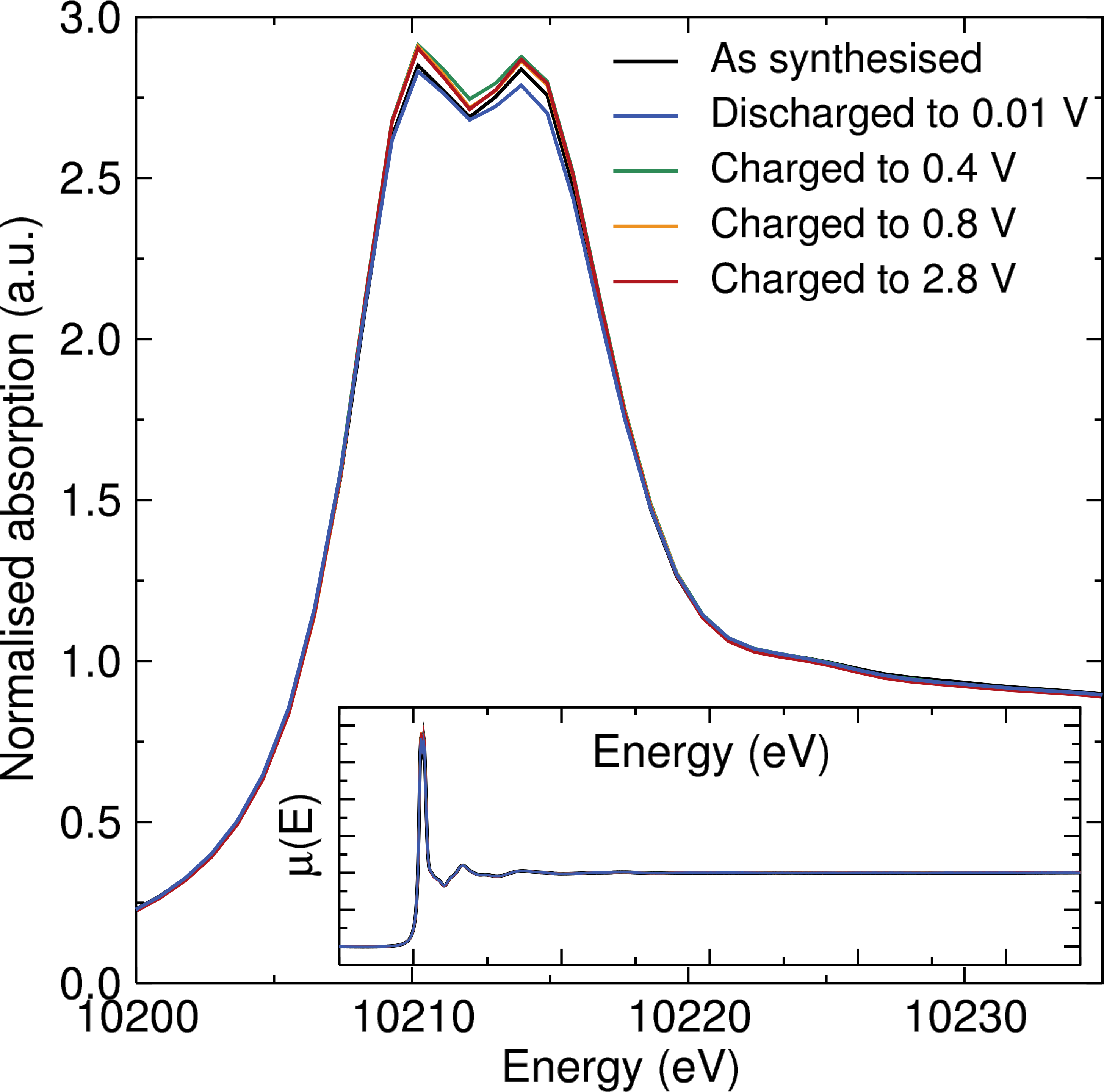


**Supplementary Figure 10:** XANES region of the XAS data obtained for W L_III_-edge (inset) for *ex-situ* samples of the Li_1.5_La_1.5_WO_6_ material cycled at different points of charge and discharge cycles. Only subtle differences on the relative intensities of the split peak can be observed.


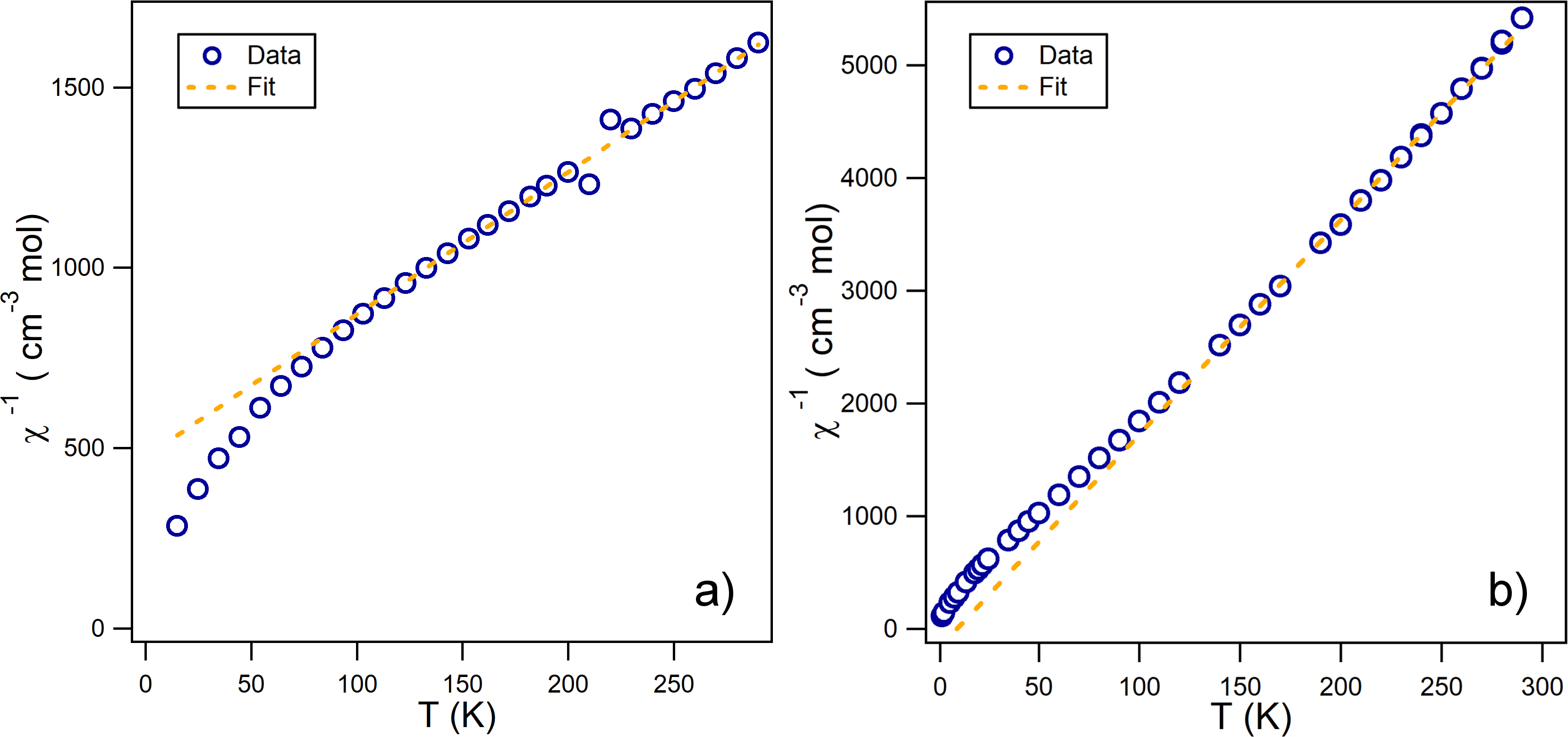


**Supplementary Figure 11:** *Ex-situ* SQUID measurements on (a) fully reduced (0.01 V) Li_1.5_La_1.5_WO_6_ material and (b) partially oxidised (0.40V) materials. The Curie-Weiss fit of the data was performed between 150 and 290 K, indicating paramagnetic behavior of *ca.* 0.8 e^-^ per formula unit of perovskite material in (a) and a greatly reduced moment indicating mostly diamagnetic material in (b).

**Supplementary Note 1**

A simulation cell with the formula Li_6_La_6_W_4_O_24_, measuring 7.87×7.89×7.87 Å and containing 8 A-sites, of which six are occupied by lanthanum and two by lithium ions, was generated to represent Li_1.5_La_1.5_WO_6_. A full enumeration of symmetry inequivalent orderings of the A-site within the simulation cell using the SOD program^3^ were generated. Due to the relative sizes of the lithium and lanthanum ions, there is a significant amount of space for the lithium ions to relax into, therefore a direct energy minimisation may not always find the most energetically favourable position for the lithium ions within the A-sites. To circumvent this, molecular dynamics calculations at 300 K using an NPT Langevin thermostat for 5 ps (0.5 fs timestep) was employed for each ordering. This allowed the lithium ions to explore configurational space from which the most visited sites can be analysed. In total, five distinct Li ion A-site orderings were identified by the SOD program, with calculations suggesting the most stable ordering is when the A-site Li ions are at nearest-neighbour sites in the x direction (NNx) in agreement with a previous study^2^ and this was used as the starting point for computationally lithiating Li_1.5_La_1.5_WO_6_. Lithium ions were inserted into the structure sequentially, targeting the largest voids and re-minimising the structure. A single point calculation using the hybrid HSE06 functional was performed on the re-minimised structure, to account for localisation of tungsten d-states and the minimized structures for LLWO as lithiated from Li_1.5_La_1.5_WO_6_ to Li_3.0_La_1.5_WO_6_ are shown in the main paper (Figure 8).

The intercalation voltages versus the number of Li ions and changes in oxidation state for the tungsten analogue were calculated using the equation^4^:

$$V=\frac{-\left[ E\left( \mathrm{Li}_{x}\mathrm{La}_{6}W_{4}O_{24} \right)-E\left( \mathrm{Li}_{y}\mathrm{La}_{6}W_{4}O_{24} \right)-\left( x-y \right)E\left( Li metal \right) \right]}{\left( x-y \right)F}$$

where, $V$ is the intercalation voltage, $x$ and $y$ are the number of lithium ions in the simulation cells, $E$ is the total energy of the defined system in brackets and $F$ is the Faraday constant. The intercalation voltages and nominal redox couples are given in Supplementary Table 6. Calculations show that the average of the W^6+^ to W^5+^ intercalation voltages is 0.76 V and the average of the W^5+^ to W^4+^ intercalation voltages is 0.18 V, in excellent agreement with the experimental CV data.

**Supplementary Table 6:** The structural formula, intercalation voltages and nominal redox couple of lithiated LLWO.

| **Structural formula** | **Number of Li ions in cell** | **# Li in A site** | **Intercalation voltage (V)** | **Nominal Redox couple** |
| --- | --- | --- | --- | --- |
| Li_1.5_La_1.5_WO_6_ | 6 | 1.0 | - |  |
| Li_1.75_La_1.5_WO_6_ | 7 | 1.5 | 0.93 | W^6+^ ---> W^5+^ |
| Li_2.0_La_1.5_WO_6_ | 8 | 2.0 | 0.60 | W^6+^ ---> W^5+^ |
| Li_2.25_La_1.5_WO_6_ | 9 | 2.5 | 0.74 | W^6+^ ---> W^5+^ |
| Li_2.5_La_1.5_WO_6_ | 10 | 3.0 | 0.77 | W^6+^ ---> W^5+^ |
| Li_2.75_La_1.5_WO_6_ | 11 | 3.5 | 0.29 | W^5+^ ---> W^4+^ |
| Li_3.0_La_1.5_WO_6_ | 12 | 4.0 | 0.08 | W^5+^ ---> W^4+^ |


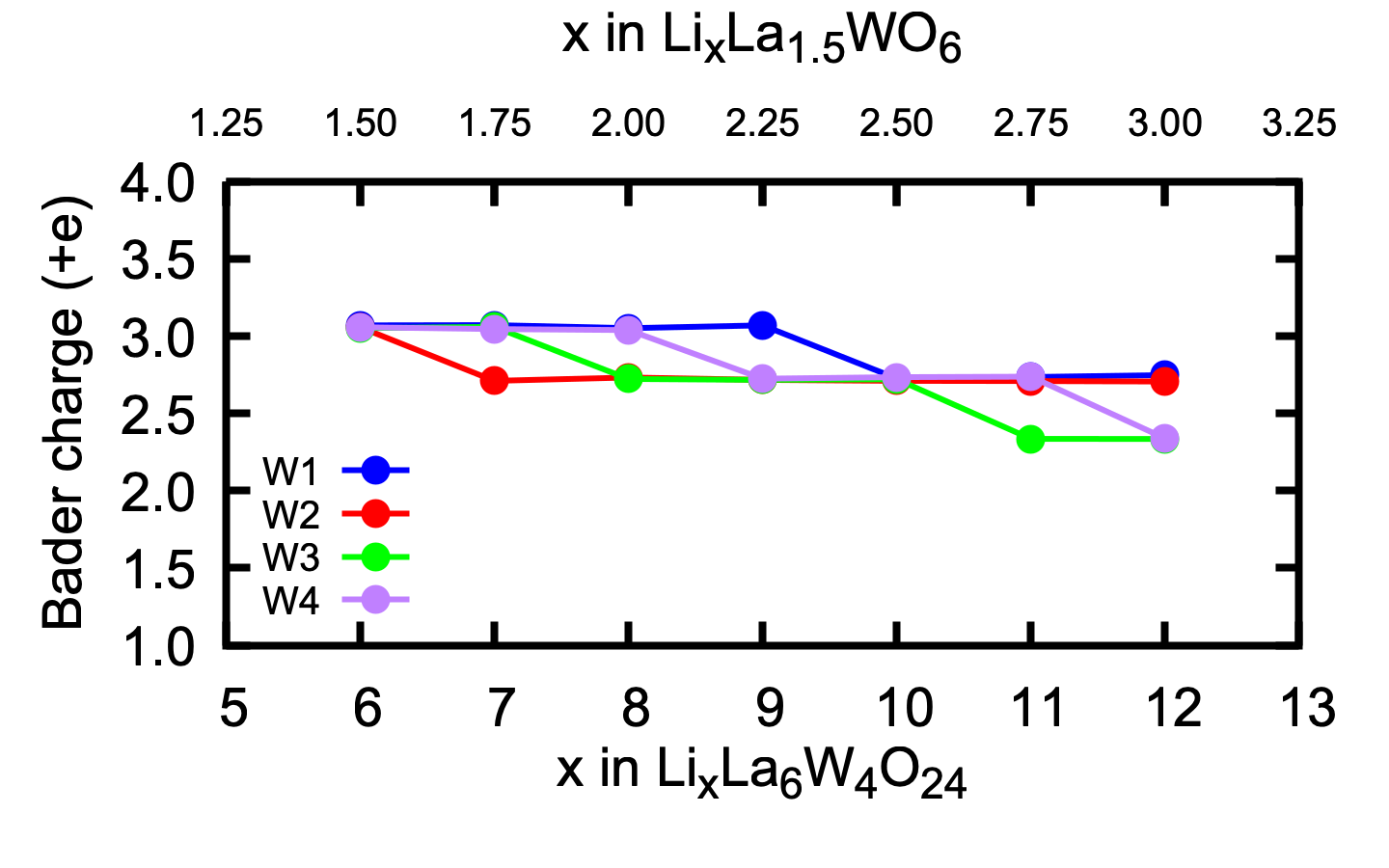


**Supplementary Figure 12:** Bader charges for tungsten calculated for computationally lithiated LLWO structure, showing a systematic reduction of all W species to 5+ before further reduction to W^4+^.

To study the redox behaviour in the Te analogues, the W cations from the optimised LLWO analogues were replaced by tellurium ions and re-minimised using the same procedure as detailed. The variation in Bader charge with Li^+^ intercalation is shown in Supplementary Figure 13, with the corresponding intercalation voltages and nominal redox couples for LLTeO listed in Supplementary Table 7. In contrast with the redox mechanism for Li^+^ intercalation on the Li_1.5_La_1.5_WO_6_ material, large intercalation voltages are found for the Li_1.5_La_1.5_TeO_6_ and reluctance of Te^6+^ to form Te^5+^, suggesting redox cycling of the Te analogue to be unlikely to have place, as experimentally observed. The W analogue, however, is capable of accessing the intermediate W^5+^ oxidation state, allowing a uniform step change in oxidation states (W^4+^ ---> W^5+^ ---> W^6+^) throughout the material during lithium deintercalation.


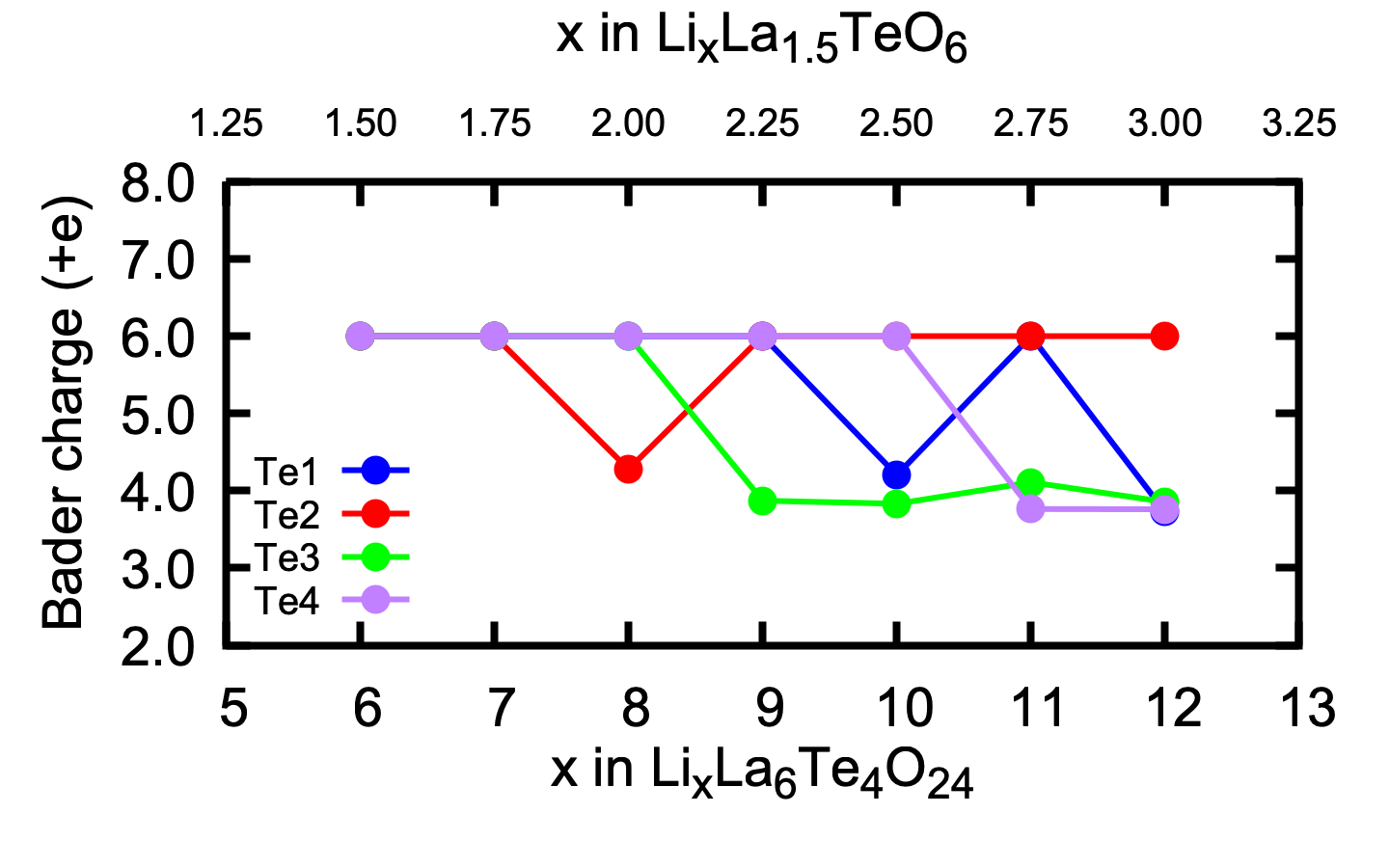


**Supplementary Figure 13:** Bader charge calculated for computationally lithiated LLTeO structures. LLTeO prefers a reduction from 6+ to 4+ before a second Te species begins reduction.

**Supplementary Table 7:** The structural formula, intercalation voltage and nominal redox couple of lithiated LLTeO.

| **Structural formula** | **Number of Li ions in cell** | **Intercalation voltage (V)** | **Nominal Redox couple** |
| --- | --- | --- | --- |
| Li_1.50_La_1.5_TeO_6_ | 6 |  |  |
| Li_1.75_La_1.5_TeO_6_ | 7 | 0.59 | Te^6+^ ---> Te^5+^ |
| Li_2.00_La_1.5_TeO_6_ | 8 | 1.64 | Te^5+^ ---> Te^4+^ |
| Li_2.25_La_1.5_TeO_6_ | 9 | 0.68 | Te^6+^ ---> Te^5+^ |
| Li_2.50_La_1.5_TeO_6_ | 10 | 1.91 | Te^5+^ ---> Te^4+^ |
| Li_2.75_La_1.5_TeO_6_ | 11 | 1.08 | Te^6+^ ---> Te^5+^ |
| Li_3.00_La_1.5_TeO_6_ | 12 | 1.53 | Te^5+^ ---> Te^4+^ |


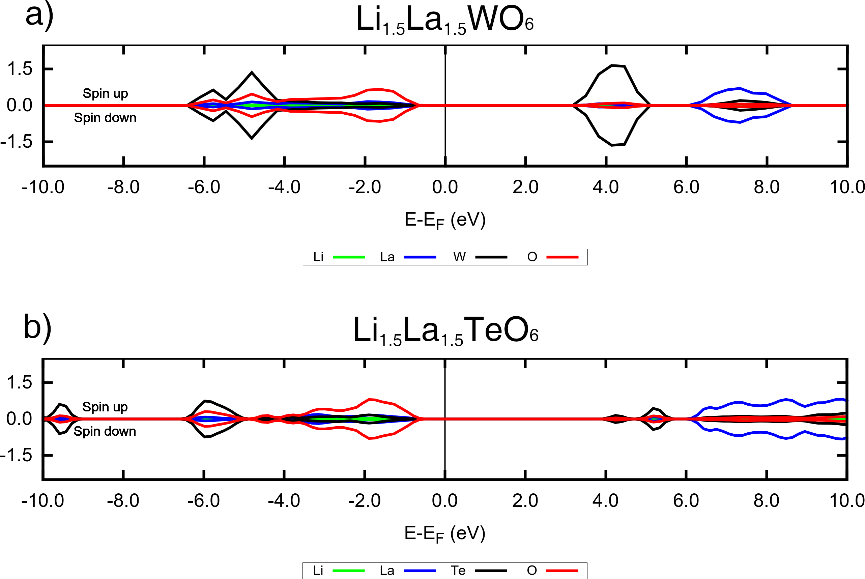


**Supplementary Figure 14:** Density of States for a) Li_1.5_La_1.5_WO_6_ and b) Li_1.5_La_1.5_TeO_6_. A clear lack of significant available states for redox reactions of the Li_1.5_La_1.5_TeO_6_ materials is observed, in contrast of those observed below 3 eV for the Li_1.5_La_1.5_WO_6_ analogue, indicating a large band gap and wide electrochemical stability window.


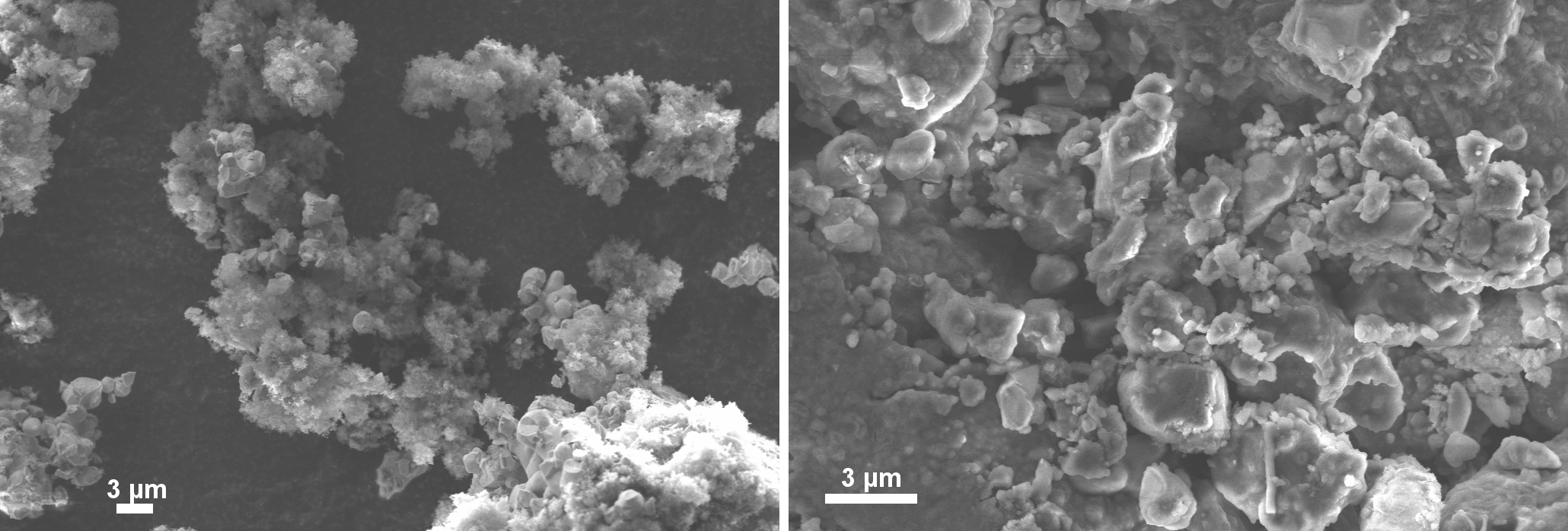


**a)**

**b)**

**Supplementary Figure 15:** SEM images of the as-prepared Li_1.5_La_1.5_WO_6_ electrode powder mixed with carbon black and PTFE (a) and cycled electrode after cell discharge to 0.01 V. The integrity of the Li_1.5_La_1.5_WO_6_ can be inferred from the particle size as it remained larger than 1 µm (see marked particles as example) as in the case of the as-prepared material. Further analyses are hampered by the large amount of SEI formed on the electrode surface.


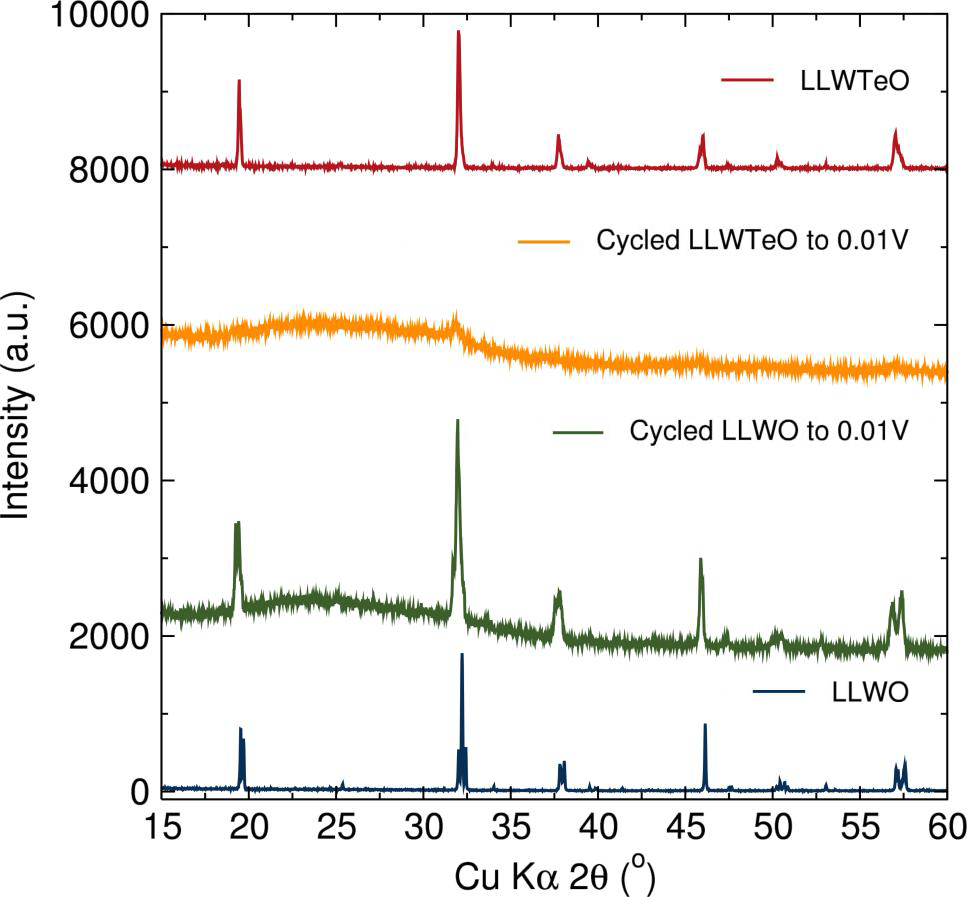


**Supplementary Figure 16**: *Ex-situ* PXRD of Li_1.5_La_1.5_WO_6_ (LLWO) and Li_1.5_La_1.5_W_0.5_Te_0.5_O_6_ (LLWTeO) materials as-synthesised and after fully reduction to 0.01 V on a Li half-cell.


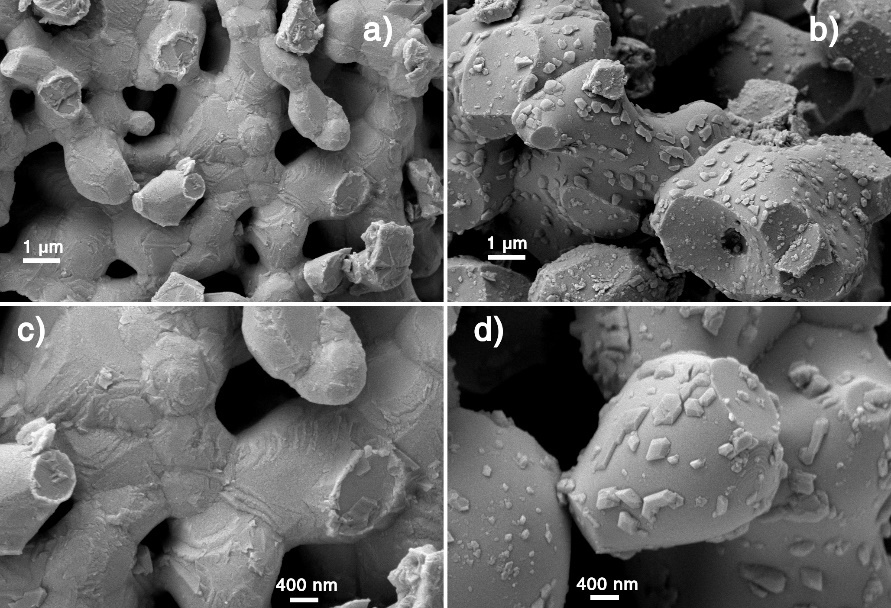


**Supplementary Figure 17:** SEM images of as-synthesised Li_1.5_La_1.5_TeO_6_ (a,c) and Li_1.5_La_1.5_WO_6_ (b, d) at different magnifications. Large bulk materials decorated with submicron-sized particles are observed for Li_1.5_La_1.5_WO_6_ perovskite. Similar bimodal particle distributions are not observed for the Li_1.5_La_1.5_TeO_6_ material.


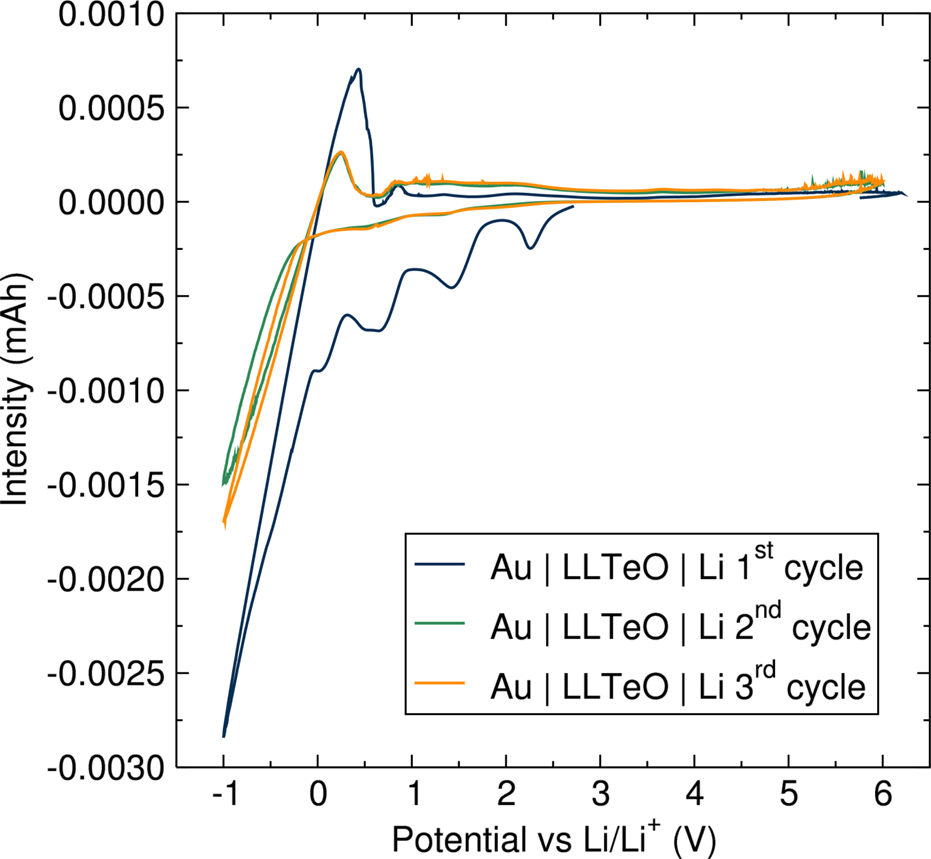


**Supplementary Figure 18:** CV measurements of the asymmetric Au|LLTeO|Li cell at 80 °C. The step rate was fixed to 0.05 mV s^-1^ and the scanning voltage range constrained to 0.01 – 6 V.

**Supplementary References**

1 A. Keren, *Phys. Rev. B*, 1994, **50**, 10039–10042.

2 A. B. Santibáñez-Mendieta, C. Didier, K. K. Inglis, A. J. Corkett, M. J. Pitcher, M. Zanella, J. F. Shin, L. M. Daniels, A. Rakhmatullin, M. Li, M. S. Dyer, J. B. Claridge, F. Blanc and M. J. Rosseinsky, *Chem. Mater.*, 2016, **28**, 7833–7851.

3 R. Grau-Crespo, S. Hamad, C. R. A. Catlow and N. H. de Leeuw, *J. Phys. Condens. Matter*, 2007, **19**, 256201.

4 F. Zhou, M. Cococcioni, C. A. Marianetti, D. Morgan and G. Ceder, *Phys. Rev. B - Condens. Matter Mater. Phys.*, 2004, **70**, 1–8.
